# Nd substitution effects on charge transport, colossal permittivity, and X6P thermal stability of Sm_2−*x*_Nd_*x*_CuO_4_ ceramics

**DOI:** 10.1039/d6ra08552k

**Published:** 2026-10-22

**Authors:** Missaoui Malek, Chouk Wael, Zouaoui Mouldi

**Affiliations:** a Faculty of Science of Bizerte, Laboratory of Physics of Materials: Structure and Property LR01ES15, University of Carthage Tunisia wael.chouk@fsb.ucar.tn mouldi.zouaoui@fsb.ucar.tn; b Faculty of Science of Bizerte, Shared Research Services Unit: Electron Microscopy (TEM) & Surface Spectroscopy, University of Carthage Tunisia

## Abstract

Temperature-stable colossal permittivity ceramics are critical for high-density energy storage and power electronics operating under demanding environments. This work systematically investigates Sm_2−*x*_Nd_*x*_CuO_4_ (*x* = 0, 0.2, 0.4) rare-earth cuprate ceramics synthesized *via* a solid-state reaction. Rietveld refinement of X-ray diffraction data confirms T′-type tetragonal structure (*I*4/*mmm*), where Nd^3+^ substitution expands the lattice and promotes grain growth without introducing secondary phases. Impedance spectroscopy and complex modulus analyses reveal that electrical transport is dominated by highly resistive grain boundaries (*α*_R_ ≈ 0.9), exhibiting a transport crossover near 290 K to oxygen vacancy-mediated Small Polaron Hoping (*E*_a_ ≈ 0.186 eV). Notably, the optimized Sm_1.6_Nd_0.4_CuO_4_ ceramic achieves a remarkable negative Temperature Coefficient of Resistance (TCR ≈ −5% K^−1^ at 390 K), highlighting its competitive potential for uncooled thermal sensing technologies. Crucially, the *x* = 0.4 composition exhibits a colossal dielectric permittivity 
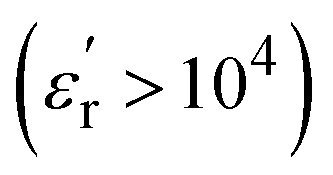
 alongside low dissipation losses, driven by an Internal Barrier Layer Capacitor (IBLC) mechanism and Maxwell–Wagner interfacial polarization. Neodymium substitution effectively suppresses thermal permittivity fluctuations, restricting relative variations to 
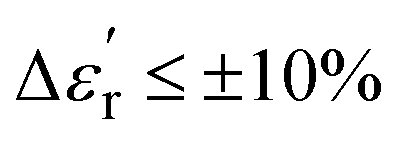
 over an extended operational window from −55 °C to 105 °C. this directly upgrades the material's thermal stability from the parent compound's X5R rating to the stringent Electronic Industries Alliance (EIA) X6P commercial significant. These superior characteristics position Sm_1.6_Nd_0.4_CuO_4_ ceramics as promising candidates for high-reliability multilayer ceramic capacitors (MLCCs) and dual-functional microelectronic devices.

## Abstract 2

Temperature-stable colossal permittivity ceramics are critical for high-density energy storage and power electronics operating under demanding environments.

## Graphical abstract

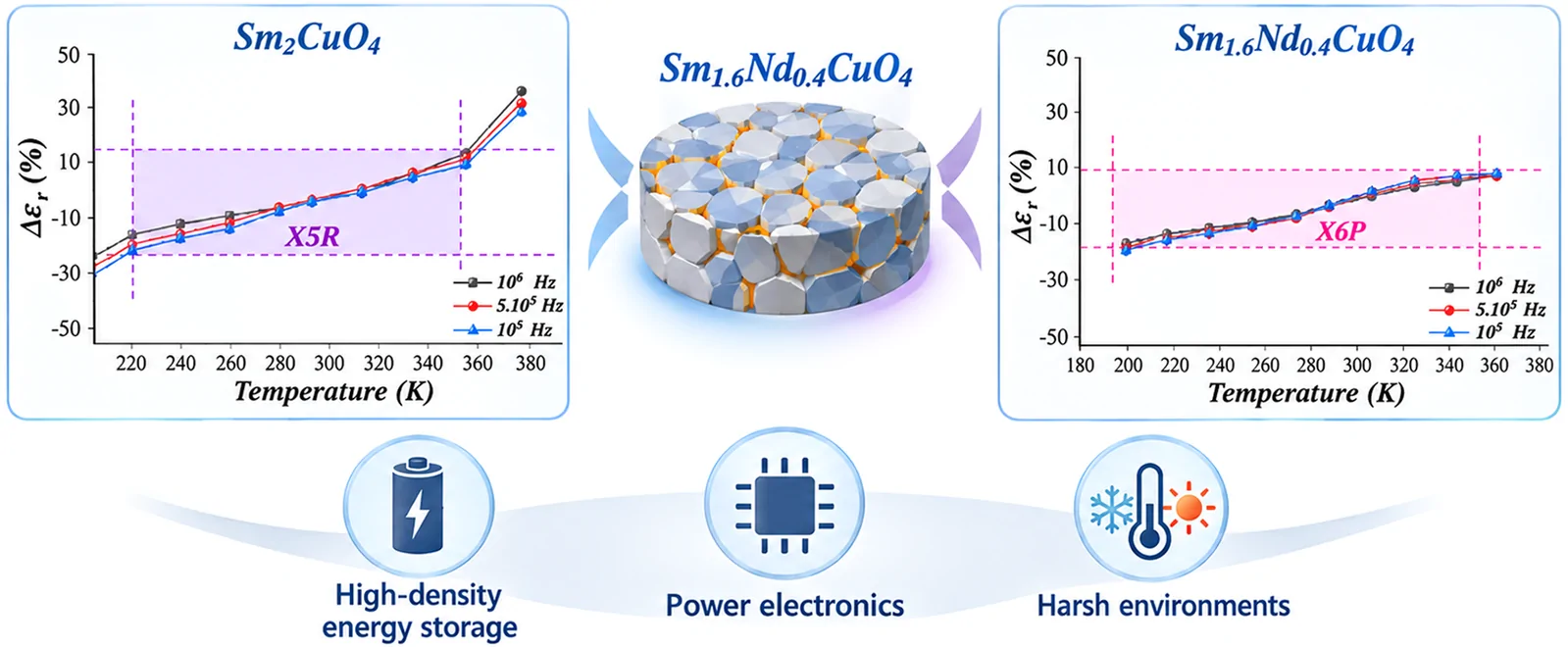

## 1. Introduction

The continuous miniaturization of electronic devices coupled with increasing demands for enhanced energy storage capabilities has intensified research into advanced dielectric materials with exceptional permittivity values.^1,2^ Among these, rare-earth cuprates with the general formula RE_2_CuO_4_(rare-earth elements) have emerged as promising candidates due to their colossal permittivity (CP) characteristics combined with remarkable structural stability.^3,4^ The present study focuses on the Sm_2−*x*_Nd_*x*_CuO_4_ system, where partial substitution of samarium with neodymium offers a strategic approach to fine-tune the structural and dielectric properties for ceramic capacitor applications.^5^

Colossal permittivity materials, characterized by high dielectric constants, represent a frontier in the development of high-energy-density capacitors.^6,7^ While traditional dielectric ceramics like BaTiO_3_ and other ferroelectric perovskites have been extensively utilized in commercial capacitors, their permittivity (a few 10^3^ at room temperature, with a maximum near the Curie temperature) varies strongly near phase transitions, and meeting the thermal stability classes of the Electronic Industries Alliance (EIA) in BaTiO_3_-based multilayer capacitors requires elaborate compositional engineering.^8,9^ The exploration of rare-earth cuprates with the K_2_NiF_4_-type structure provides an alternative pathway toward materials with substantially higher permittivity values and a stable permittivity over a wide temperature range, the dielectric loss depending strongly on frequency.^10,11^

The thermal stability of capacitors is classified by the EIA RS-198 standard through a three-character code.^12^ The first letter gives the lower limit of the operating temperature (*X*: −55 °C), the digit gives the upper limit (5: +85 °C, 6: +105 °C, 7: +125 °C), and the last letter gives the maximum variation of the capacitance, referred to its value at 25 °C, over this range (*R*: ±15%, *P*: ±10%). A class X5R capacitor therefore keeps its capacitance within ±15% between −55 and +85 °C, whereas an X6P capacitor must remain within ±10% between −55 and +105 °C, a more demanding specification both in temperature range and in tolerance. These classes are those of the multilayer ceramic capacitors used in portable electronics and power electronics, so that they provide a direct, application-oriented measure of the thermal stability of a new dielectric. Colossal permittivity cuprates from our group have already been evaluated against this standard: GdCa_2_Cu_3_O_*δ*_ (Y5R/Y6R),^13^ Sm_2_CuO_4_ (X5R)^14^ and Gd_1.3_Sr_1.7_Cu_2_O_*δ*_ (X7R/X7P).^15^ In the present work, we investigate whether the partial substitution of Sm by Nd can improve the thermal stability of Sm_2_CuO_4_ from the X5R to the X6P class.

The parent compound Sm_2_CuO_4_ has previously demonstrated impressive dielectric properties with permittivity values of the order of 10^3^–10^4^, depending on frequency, at room temperature, and a temperature stability corresponding to the X5R class.^14^ However, systematic investigations into the effects of A-site cation substitution on both structural and dielectric characteristics remain limited.^16^ Neodymium substitution is particularly interesting due to the similar ionic radius, but different electronic configuration compared to samarium, potentially affecting the crystal field interactions, electronic band structure, and consequently, the polarization mechanisms responsible for the colossal permittivity.^17,18^

This study presents a comprehensive investigation of the Sm_2−*x*_Nd_*x*_CuO_4_ system across a range of compositions (0 ≤ *x* ≤ 0.4), with particular emphasis on correlating structural parameters with dielectric responses. We employ a multi-technique approach combining X-ray diffraction (XRD),^19^ transmission electron microscopy (TEM),^20^ and impedance spectroscopy^21^ to elucidate the fundamental structure–property relationships in these materials. The temperature and frequency dependence of dielectric properties are systematically analysed to identify the underlying polarization mechanisms and assess their relevance for practical capacitor applications.^22,23^

The implications of our findings extend beyond fundamental materials science to address practical challenges in electronic component design.^24^ By establishing composition-structure–property relationships in the Sm_2−*x*_Nd_*x*_CuO_4_ system. Our goal is to create regulations for controlled cation substitution that will allow us to modify dielectric characteristics.^25^ The investigation of thermal stability, frequency response, and energy storage characteristics provides critical insights for evaluating the potential of these materials in next-generation ceramic capacitors capable of operating under demanding conditions while offering enhanced energy storage density.^26,27^ Furthermore, understanding the localized charge transport kinetics and temperature-dependent resistance offers a pathway to evaluate this cuprate system for highly sensitive uncooled infrared bolometers and advanced thermal sensing technologies.

## 2. Experimental details

Sm_2−*x*_Nd_*x*_CuO_4_ (*x* = 0, 0.2, 0.4) ceramic samples were synthesized *via* the conventional solid-state reaction method, following the same synthesis protocol that we previously reported for the parent compound Sm_2_CuO_4_.^14^ High-purity Sm_2_O_3_, Nd_2_O_3_, and CuO powders (≥99.99%) were weighed with an accuracy of 10^−4^ g in the appropriate stoichiometric proportions to achieve the targeted Nd^3+^ substitution levels on the Sm site, with Nd_2_O_3_ introduced as the additional precursor required for this substitution. The precursors were thoroughly mixed in an agate mortar, granulated, and pressed into pellets. These pellets were calcined at 950 °C for 12 h in open air using a heating rate of 10°C min^−1^. The resulting pellets were then reground, recompacted, and sintered twice at 950 °C, each for 12 h. After each calcination or sintering step, the samples were slowly cooled to room temperature under ambient air.

The synthesized Sm_2−*x*_Nd_*x*_CuO_4_ ceramics were comprehensively characterized using multiple analytical techniques. Phase identification and crystal structure analysis were performed using a Bruker D8 Advance X-ray diffractometer equipped with CoK_α_ radiation (*λ* = 1.789 Å), scanning over a 2*θ* range of 10–90°. Rietveld refinement analysis was conducted using the Fullprof software package to determine precise structural parameters and phase quantification, allowing us to monitor the *c*-axis parameter, which serves as a highly sensitive indicator of oxygen stoichiometry. The surface chemical composition, oxygen defect concentration, and resulting oxidation states were analysed *via* X-ray photoelectron spectroscopy (XPS) at room temperature using an Omicron spectrometer with a non-monochromatized AlK_α_ (*hυ* = 1486.6 eV) source. XPS survey scans ranged from 5 to 1350 eV spectra were collected, utilizing a 50 eV pass energy and 0.5 eV resolution steps for each scan. Microstructural investigation of the sintered ceramics was carried out using transmission electron microscopy (TEM) to examine grain morphology, size distribution, and interfacial characteristics. Complex impedance spectroscopy (CIS) measurements were performed to characterize the dielectric properties across a broad frequency range (10 Hz–13 MHz) and various temperatures, enabling comprehensive analysis of the dielectric behavior of the synthesized perovskite ceramics.

## 3. Results and discussion

### 3.1 Crystal structure

The X-ray powder diffractogram of the synthesised ceramic compounds Sm_2−*x*_Nd_*x*_CuO_4_ is displayed in Fig. 1a. The diffractogram shows no peaks corresponding to reactants or intermediate compounds, indicating the successful synthesis of a single-phase solid solution within the detection limit of XRD. Furthermore, the Miller indices (*hkl*) align precisely with established literature ref. 14.

**Fig. 1:**
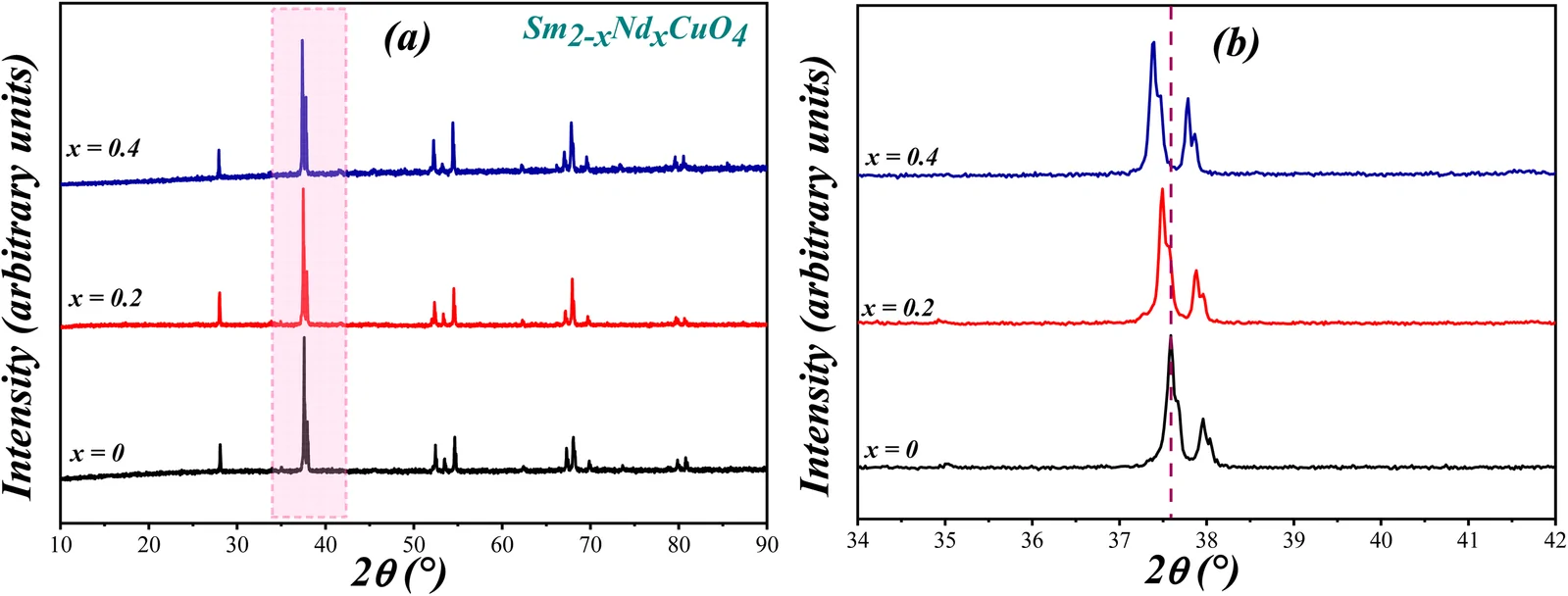
XRD patterns of Sm_2−*x*_Nd_*x*_CuO_4_ ceramics (*x* = 0, 0.2, 0.4), (a) full XRD patterns, (b) partially magnified patterns.

XRD patterns of as-sintered Sm_2−*x*_Nd_*x*_CuO_4_ ceramics for varying Nd-cation contents are displayed in Fig. 1b. A minor shift of the diffraction peaks toward lower 2*θ* angles occurs when Nd is incorporated into the Sm_2_CuO_4_ matrix, consistent with lattice expansion. The ionic size differences between Nd^3+^ (0.98 Å) and Sm^3+^ (0.96 Å) perturb the crystal structure,^18^ expanding the lattice parameters (Table 1) and altering the physicochemical characteristics. Additionally, the differing electronic configurations of Nd ([Xe] 4f^4^ 6s^2^) and Sm ([Xe] 4f^6^ 6s^2^) can modify electrostatic interactions and electronic states near the Fermi level, which is crucial for influencing the material's transport, electrical and magnetic properties.^28^ Substitution of Sm by Nd leads to subtle structural modifications that affect the lattice parameters and ionic interactions, an essential consideration for applications in functional materials and development of new conductors.^29,30^

**Table 1:** Structural parameters of Sm_2−*x*_Nd_*x*_CuO_4_ samples deduced by Rietveld refinement from XRD data

Sm_2−*x*_Nd_*x*_CuO_4_	*x* = 0					*x* = 0.2					*x* = 0.4				
Crystal structure	Tetragonal														
Space group	*I*/4*mmm*														
	Atom	*x*	*y*	*z*	Occupancy	Atom	*x*	*y*	*z*	Occupancy	Atom	*x*	*y*	*z*	Occupancy
	Sm1	0	0	0.3518(2)	1.9782	Sm	0	0	0.3521(2)	1.7859	Sm	0	0	0.3527(7)	1.5967
	Sm2	0	0	0.3518(2)	0	Nd	0	0	0.3521(2)	0.1985	Nd	0	0	0.3527(7)	0.4042
	Cu	0	0	0	0.9885	Cu	0	0	0	0.9887	Cu	0	0	0	0.9886
	O1	0	0.5	0	2.0375	O1	0	0.5	0	2.0154	O1	0	0.5	0	1.9999
	O2	0	0.5	0.25	2.0543	O2	0	0.5	0.25	2.0498	O2	0	0.5	0.25	2.0399
Lattice parameters	*a* = *b* = 3.912(5) Å					*a* = *b* = 3.917(5) Å					*a* = *b* = 3.919(0) Å				
	*c* = 11.970(3) Å					*c* = 11.990(3) Å					*c* = 11.996(8) Å				
	*α* = *β* = *γ* = 90°					*α* = *β* = *γ* = 90°					*α* = *β* = *γ* = 90°				
Goodness of fit	*χ* ^2^ = 1.42					*χ* ^2^ = 1.43					*χ* ^2^ = 1.54				

Rietveld refinement was performed using the Fullprof software to determine the structural parameters, atomic positions, and site occupancies of the Sm_2−*x*_Nd_*x*_CuO_4_ ceramics. The refinement procedure began with background fitting using a cosine function, followed by peak-profile fitting using pseudo–Voigt functions. The refined parameters included the background coefficients, scale factor, zero shift, lattice parameters (*a* and *c*), atomic positional coordinates (*x*, *y*, and *z*), isotropic atomic displacement parameters, profile half–width parameters (*u*, *v*, and *w*), preferred orientation, and atomic site occupancies. The oxygen-site occupancies were refined with an upper bound of 1 to estimate the oxygen deficiency in the crystal structure. The refined structural models showed good agreement between the observed and calculated diffraction patterns (Fig. 2a–c). The corresponding values are reported in Table 1 and indicate satisfactory agreement between the experimental and calculated patterns. Fig. 2 presents the Fullprof refinement results.

**Fig. 2:**
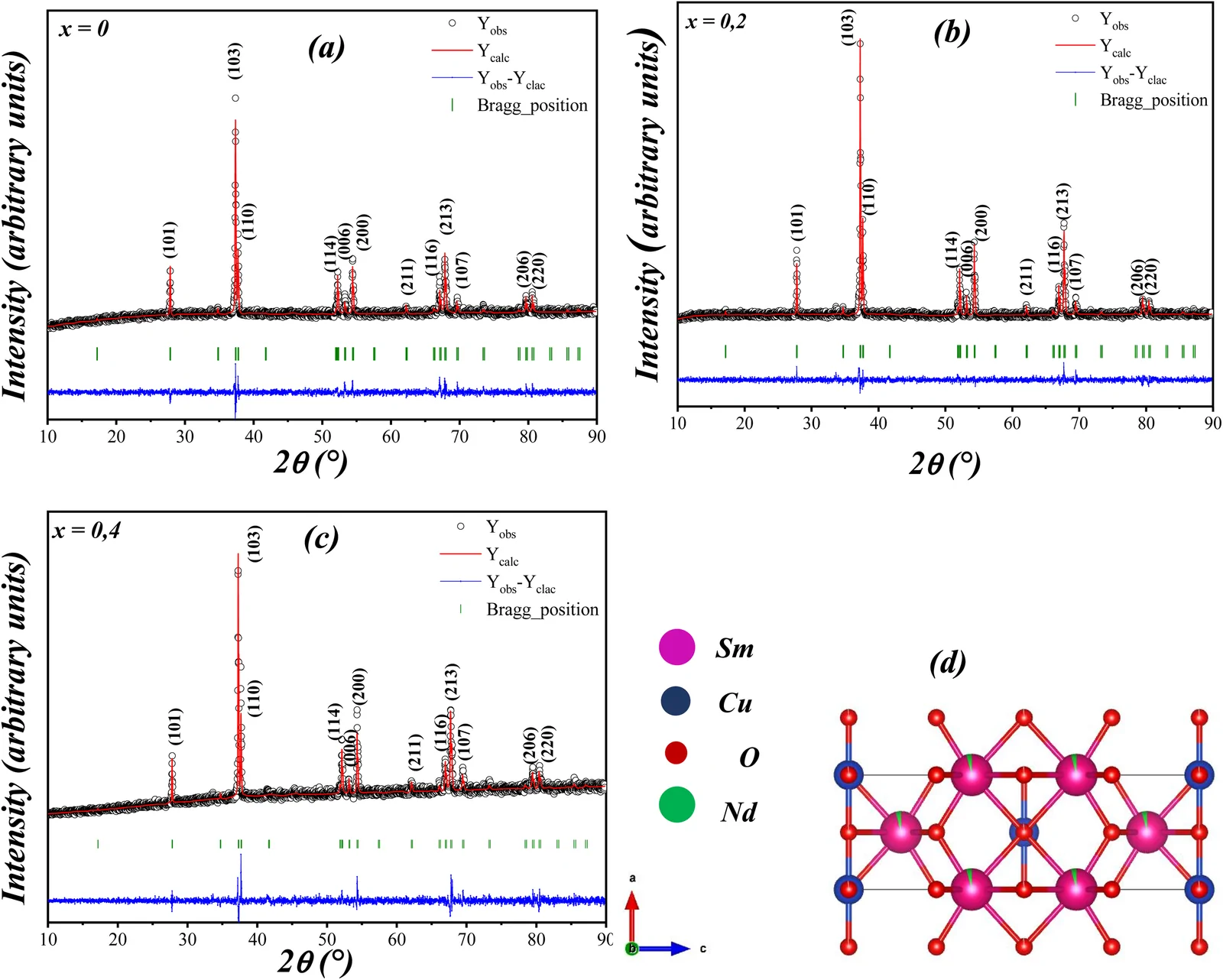
XRD patterns and Rietveld refinement of Sm_2_CuO_4_ ceramics (a), Sm_1.8_Nd_0.2_CuO_4_ ceramics (b), Sm_1.6_Nd_0.4_CuO_4_ ceramics (c)); with the red line the calculated, Black line observed, blue line difference between the two previous ones, and Bragg position, and (d) crystallographic structure of compound.

All diffractograms retain the characteristic reflection pattern of the parent compound. All observed reflections could be indexed within the same tetragonal space group, *I*4/*mmm*, with no additional reflections attributable to detectable secondary or impurity phases. These results indicate that Nd substitution preserves the average tetragonal structure within the detection limits of the X-ray diffraction measurements. The refined structural parameters are summarized in Table 1.

The oxygen stoichiometry was further evaluated from the refined oxygen-site occupancies obtained by Rietveld analysis. For the tetragonal *I*4/*mmm* structure, the oxygen deficiency parameter, *δ*, was estimated from the refined occupancies. Considering the two oxygen sites, O1 and O2, each with a multiplicity of four, and two formula units per unit cell, we calculated the oxygen content per formula unit from the sum of contributions from the crystallographic oxygen sites. The estimated oxygen deficiency values are approximately 0.0163, 0.033, and 0.039 for *x* = 0, 0.2 and 0.4, respectively. These values correspond to the estimated compositions Sm_2_CuO_3.984_, Sm_1.8_Nd_0.2_CuO_3.967_, and Sm_1.6_Nd_0.4_CuO_3.961_, respectively. The progressive decrease in the estimated oxygen content with increasing Nd concentration points to a slight apparent increase in oxygen deficiency, which may influence the charge transport and dielectric response of the materials. These structural estimates are discussed in conjunction with the XPS results and dielectric measurements in the following sections to explore the potential role of oxygen-related defects in the observed electrical and dielectric properties. Nevertheless, because X-ray scattering from oxygen is weak in the presence of the heavy Sm and Nd atoms, the refined oxygen occupancies carry relatively large uncertainties and should be regarded as indicative trends rather than quantitative measurements of the absolute oxygen content.

The refined crystal structure model for the Sm_2−*x*_Nd_*x*_CuO_4_ series is shown alongside the corresponding atomic legend (Sm, Cu, O, Nd) in Fig. 2d. The structure adopts the characteristic *T*′-type tetragonal arrangement,^31^ built from alternating [Cu–O] planes and [Sm, Nd–O] layers stacked along the *c*-axis in a staggered configuration. The partial substitution of Sm by Nd takes place on the rare-earth site without modification of this layered architecture, as indicated by the distribution of Nd atoms among the Sm sites in the refined model.

Several analytical methods are commonly used to estimate the crystallite size and microstructural parameters from X-ray diffraction data. The structural parameters extracted from these models are summarized in Table 2. The simplest and most widely employed approach is the Debye–Scherrer equation,^32^ which relates the average crystallite size (*D*) to the broadening of a single diffraction peak:
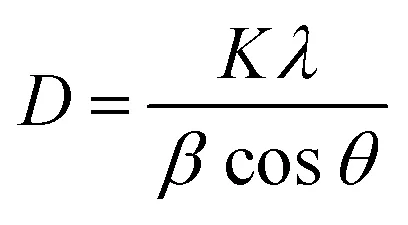
where *λ* is the X-ray wavelength used, *β* is the full width at half maximum (FWHM) of the diffraction line expressed in radians, *K* is the shape factor (typically ∼0.9), and *θ* is the corresponding diffraction angle. This method assumes that peak broadening originates solely from the finite size of the crystallites.

**Table 2:** Crystallite sizes, stress and dislocation density of samples Sm_2−*x*_Nd_*x*_CuO_4_

Sm_2−*x*_Nd_*x*_CuO_4_	Debye–Scherrer	Williamson–Hall		
	*D* (nm)	*D* (nm)	*ς*	*δ*
*x* = 0	123.39	318.45	6.35 × 10^−4^	3.99 × 10^−7^
*x* = 0.2	384.23	410.38	4.16 × 10^−4^	5.93 × 10^−6^
*x* = 0.4	530.76	734.99	5.30 × 10^−4^	1.14 × 10^−6^

It is well established that lattice strain (*ς*) arising from defects and distortions within the crystal lattice also contributes to peak broadening. The strain contribution can be approximated independently using the Stokes–Wilson relation, 
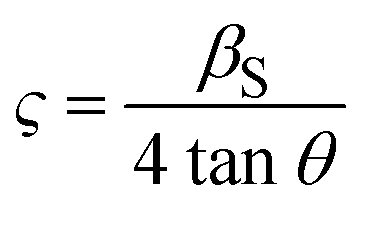
. To overcome these limitations of the Scherer equation, the Williamson–Hall method offers a more complete analysis by combining the contributions of crystallite size and lattice strain into a single linear relation: 
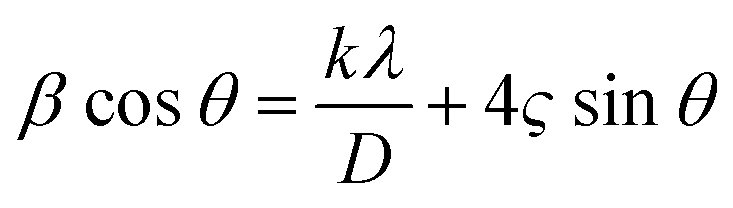
. In this framework, peak broadening is attributed jointly to size-dependent term and a strain-dependent term, which vary differently with the diffraction angle *θ*.

As the Nd content increases from *x* = 0 to *x* = 0.4, the crystallite size (*D*) increases progressively from 0.12 to 0.53 µm (Debye–Scherrer) and from 0.32 to 0.73 µm (Williamson–Hall). Since all samples were sintered under identical conditions (950 °C), this trend is attributed to the Nd substitution. The larger ionic radius of Nd^3+^ compared to Sm^3+^, expands the lattice and increases local distortion. The lattice strain (*ς*) remains of the same order of magnitude (∼4−6 × 10^−4^ for all compositions, while the dislocation density (*δ*) increases from 3.99 × 10^−7^ nm^−2^ for *x* = 0 to 1.14 × 10^−5^ nm^−2^ for *x* = 0.4, indicating a progressive accumulation of crystalline defects. Since lattice distortion and defects influence cation diffusion and grain-boundary mobility,^33–35^ they may facilitate crystallite coalescence during sintering. This interpretation is consistent with the dependence of microstructure on ionic radius reported in ReBa_2_Cu_3_O_*y*_ ceramics.^33^ However, grain growth also depends on sintering temperature, time, and atmosphere, and the effect of dopants is system-dependent;^34,36^ this mechanism should therefore be regarded as a plausible explanation rather than a confirmed one.

### 3.2 X-ray photoelectron spectroscopy (XPS) analyses

X-ray photoelectron spectroscopy (XPS) was performed to investigate the chemical composition, oxidation states, and surface characteristics of Sm_2−*x*_Nd_*x*_CuO_4_ (*x* = 0, 0.2, 0.4) ceramics.

#### 3.2.1 Survey spectra and elemental composition

Typical broad energy scans of the as-prepared ceramics are presented in the survey spectra (Fig. 3a). These scans, collected over a wide binding energy (BE) range, confirm the presence of all expected fundamental elements corresponding to their specific electron orbitals: samarium (Sm 3d), copper (Cu 2p), and oxygen (O 1s) for the parent compound. As the doping level increases to *x* = 0.2 and *x* = 0.4, the Nd 3d signals appear and grow progressively stronger, accompanied by a corresponding decrease in the Sm 3d intensity. This provides direct spectroscopic evidence of the successful partial substitution of Sm^3+^ by Nd^3+^ in the crystal lattice, with no detectable impurity phases. A residual peak related to minor surface contamination is also observed, corresponding to adventitious carbon (C 1s) at a binding energy of ∼285.0 eV.^37^ The precise position of this C 1s peak was utilized as a standard internal reference to correct for charge shift and calibrate the binding energy scale for all recorded spectra. To accurately determine the elemental composition and oxidation states, the registered high-resolution spectra for individual elements were subjected to a Shirley background subtraction and subsequently fitted using Gaussian profiles.

**Fig. 3:**
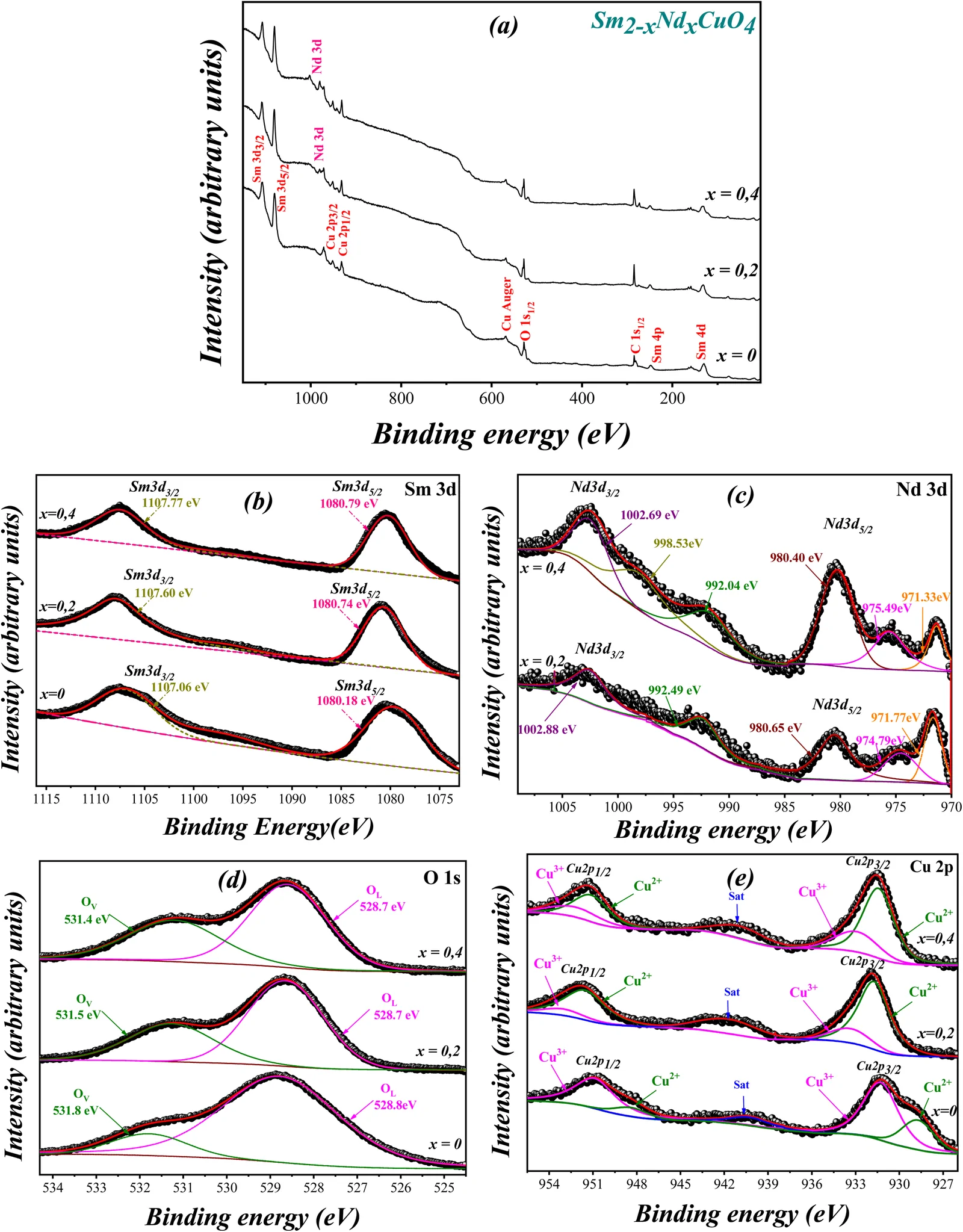
(a) Photoemission intensity distribution (survey spectra) of Sm_2−*x*_Nd_*x*_CuO_4_; high-resolution XPS peaks with multiple curve-fittings for (b) Sm (3d); (c) Nd (3d) (d) O (1s) and (e) Cu (2p).

#### 3.2.2 Rare-earth elements (Sm d and Nd 3d)

The substitution mechanism is further validated by high-resolution scans of the rare-earth elements. The Sm 3d Spectra (Fig. 3b) exhibit the characteristic spin–orbit doublet, with Sm 3d_5/2_ and Sm 3d_3/2_ peaks located at ∼1080 eV and ∼1107 eV, respectively. Along with their associated satellite features, these peaks confirm that samarium exists entirely in the Sm^3+^ oxidation state throughout the series.^38^ Similarly, the Nd 3d spectra (Fig. 3c) display well-resolved Nd 3d_5/2_ (∼980 eV) and Nd 3d_3/2_ (∼1002 eV) peaks with shake-up satellites, verifying that neodymium is incorporated exclusively as Nd^3+^. Because Nd^3+^ ion (0.98 Å) is slightly larger than Sm^3+^ (0.96 Å) in eight-fold coordination, its incorporation into the A-site introduces local lattice strain and cationic disorder around the CuO_2_ planes without modifying the formal oxidation state of the dopant itself.

#### 3.2.3 Oxygen (O 1s) states and surface defects

The structural distortions caused by this ionic size mismatch strongly influence the oxygen environment. The O 1s spectra (Fig. 3d) was deconvoluted into two main components. The low-binding-energy peak (O_L_, ∼528.7–528.8 eV) is attributed to lattice oxygen (O^2−^) in metal–oxygen bonds. The higher-binding-energy component (O_V_, ∼531.4–531.8 eV) is associated with surface oxygen species, including oxygen vacancies, hydroxyl groups, and adsorbed oxygen.^37^ Notably, the relative intensity of the O_V_ component increases with Nd content, shifting from *x* = 0 (O_L_ at 528.8 eV, O_V_ at 531.8 eV) to *x* = 0.2 (O_L_ at 528.7 eV, O_V_ at 531.5 eV) and *x* = 0.4 (O_L_ at 528.7 eV, O_V_ at 531.4 eV). This evolution indicates a growing concentration of surface defects driven by the Nd^3+^/Sm^3+^ size mismatch, which induces local structural distortions.^39^ Since the O_V_ component also contains hydroxyl groups and adsorbed species, and since XPS probes only the first nanometres of the surface, the O_V_/O_L_ ratio is a qualitative indicator of the surface oxygen-vacancy concentration and not a measure of the bulk oxygen content.

#### 3.2.4 Copper (Cu 2p) oxidation states

The Cu 2p spectrum (Fig. 3e) displays the spin–orbit doublet alongside a prominent shake-up satellite peak at ∼940 eV. This satellite is a primary diagnostic feature for the Cu^2+^ oxidation state and confirms the absence of Cu^+^ species, which is consistent with typical reports for perovskite-type ceramic materials.^40^ However, deconvolution further reveals a Cu^3+^ component whose relative intensity decreases as the Nd content increases. This shift can be attributed to structural distortions in the Cu–O–Cu bond angle induced by the larger Nd^3+^ ions, resulting in a progressive increase in the Cu^2+^ fraction within the Sm_2−*x*_Nd_*x*_CuO_4_ matrix.

Based on our previous research on copper perovskite-based ceramics,^24,41^ both XPS and XRD analyses indicate that high concentrations of Cu^3+^ in the tetragonal phase primarily arise from an order-to-disorder transition of oxygen atoms within the basal planes. Several experimental studies similarly report the presence of Cu^3+^ ions in copper oxide ceramics at room temperature.^42,43^ These studies suggest that while oxygen holes tend to localize on Cu–O pairs at high temperatures, they are more likely to associate with individual Cu–O atoms at room temperature. This transition between the two electronic configurations can be represented by the following electronic reaction:^24^

1 Cu^2+^ + O^−^ ↔ Cu^3+^ + O^2−^

Ultimately, these XPS results confirm the successful incorporation of Sm^3+^, Nd^3+^, and Cu^2+^ ions in the matrix with their expected oxidation states. Importantly, the systematic enhancement of surface oxygen vacancies observed *via* XPS is in excellent agreement with the structural estimations of the oxygen deficiency obtained from the XRD Rietveld refinement (Table 1). As all samples were prepared under identical conditions (open air, same thermal cycles), these compositional trends are highly meaningful and have significant potential implications for the magnetic, transport, and catalytic properties of this cuprate system.^44–46^

### 3.3 Microstructural analysis *via* TEM and EDX

Transmission electron microscopy (TEM) and energy-dispersive X-ray spectroscopy (EDX) were employed to investigate the morphology, local composition, and microstructural evolution of the synthesized ceramics.^14,20^ Specifically, this analysis compares the parent compound (Sm_2_CuO_4_, *x* = 0) with the heavily substituted sample (Sm_1.6_Nd_0.4_CuO_4_, *x* = 0.4)) to determine how Nd incorporation influences the material's structural architecture at the micrometer scale.

The conventional TEM micrographs (Fig. 4a and 5a) display the micrometer-scale grain morphology of the *x* = 0 and *x* = 0.4 samples, respectively. Coupled with Selected Area Electron Diffraction (SAED) (Fig. 4b and 5b), this technique allows for the precise identification of local crystallographic orientations and transition zones between grains. Analysing these transition zones between individual grains is essential, as they critically govern the material's electrical conduction pathways.

**Fig. 4:**
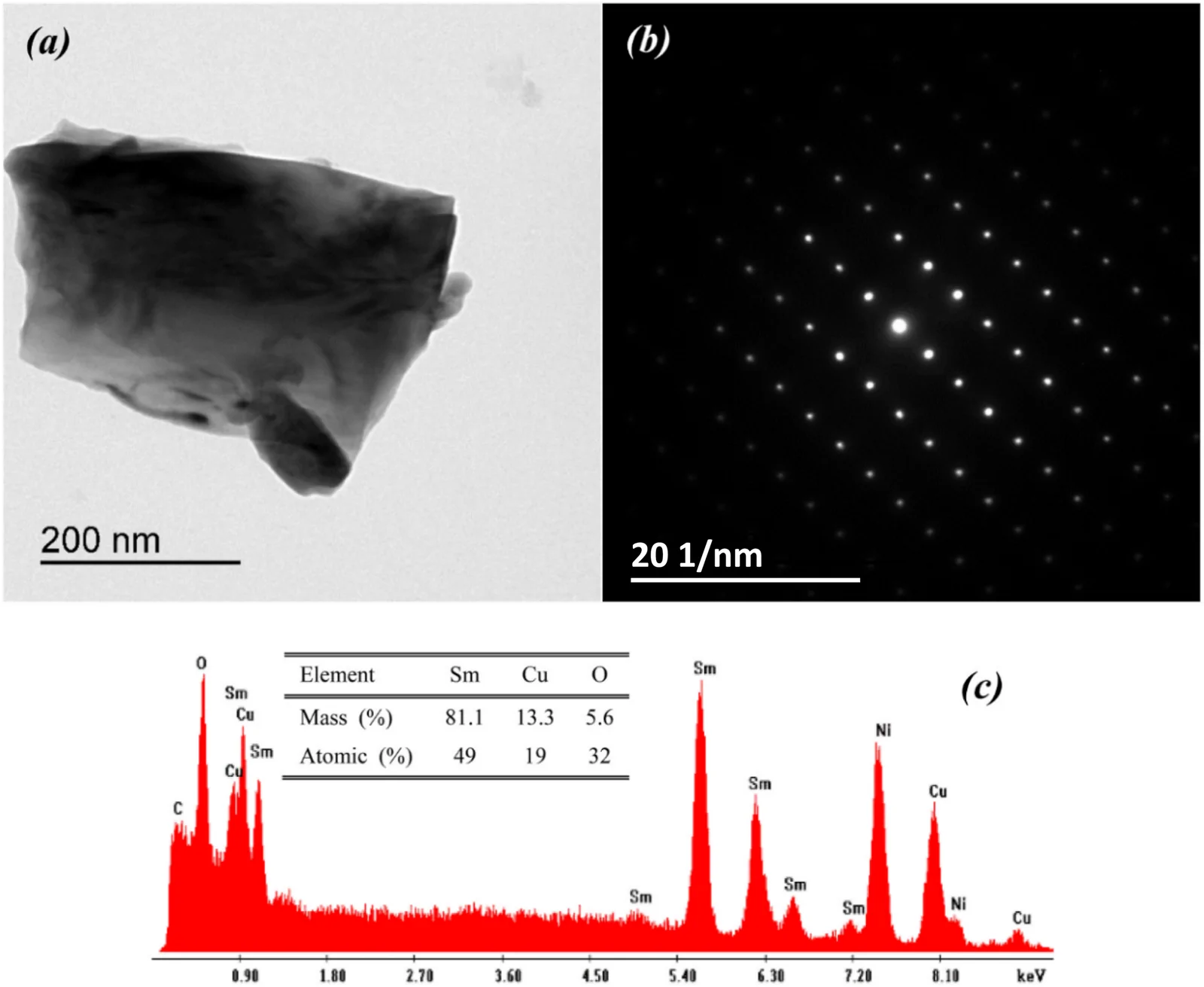
Microstructural characterization of the parent Sm_2_CuO_4_ (*x* = 0) ceramic: (a) conventional TEM image showing the micrometer-scale grain morphology, (b) corresponding selected area electron diffraction (SAED) pattern, and (c) EDX spectrum with quantitative elemental composition.

**Fig. 5:**
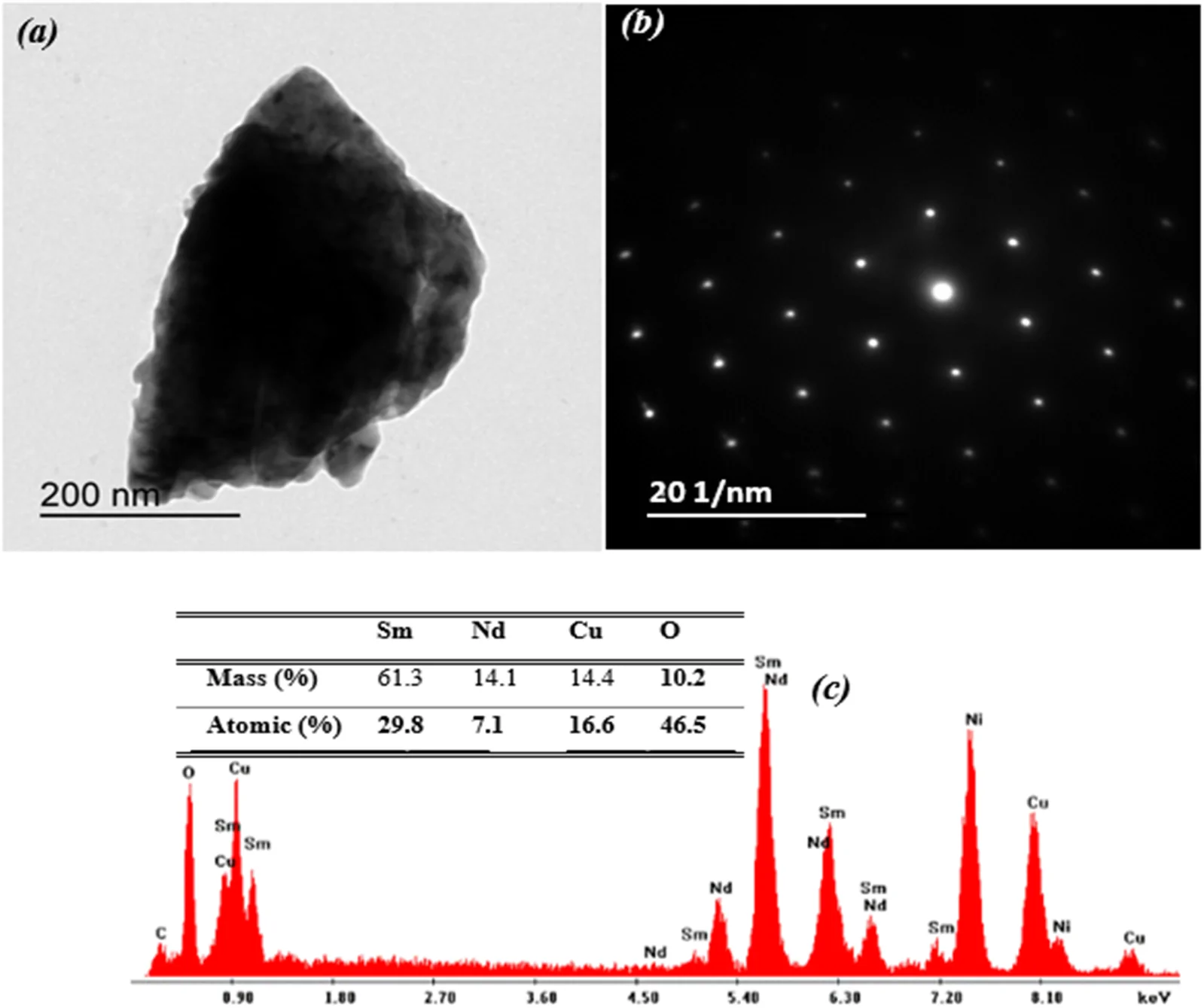
Microstructural characterization of the Nd-doped Sm_1.6_Nd_0.4_CuO_4_ (*x* = 0.4) ceramic: (a) conventional TEM image, (b) corresponding SAED pattern, and (c) EDX spectrum confirming the successful incorporation of the Nd into the ceramic matrix.

The localized chemical compositions were verified using EDX microanalysis, with the corresponding spectra and elemental distribution tables shown in Fig. 4c and 5c the parent sample (*x* = 0) exhibits the expected characteristic peaks for Sm, Cu and O. Conversely, the substituted sample (*x* = 0.4) clearly demonstrates the emergence of Nd peaks alongside the host elements. The calculated atomic and mass percentages strongly align with the intended stoichiometric ratios of the syntheses. Note that the prominent Carbon and Nickel signals present in the spectra are standard artefacts originating from the carbon-coated nickel grid used to support the samples during analysis, and have been rightly excluded from the quantitative ceramic composition.

Correlating these TEM and EDX findings with the earlier XRD results confirms that the partial substitution of Sm by Nd was highly successful and occurred without inducing drastic microstructural degradation. However, a key observation is that Nd substitution appears to promote the formation of extended monodomains. Consequently, this leads to a notable reduction in grain boundary density, a microstructural refinement that is anticipated to be a pivotal factor in dictating the transport and electrical properties of these doped materials.

### 3.4 Dielectric and complex impedance analysis

#### 3.4.1 Compositional screening and optimization strategy

Probing the dielectric response of perovskite cuprate ceramics offers a profound diagnostic window into the dynamics of locally confined charge carriers. This approach yields critical insights into fundamental electronic phenomena, including dielectric relaxation kinetics, microscopic polarization mechanisms, and the intrinsic pathways governing electrical transport. The macroscopic dielectric behavior of these perovskite-structured oxides is typically dictated by a multi-parametric interplay of operating frequency, thermal environment, microstructural surface morphology, and local stoichiometry.

To decouple and quantify these discrete contributions, alternating current (AC) impedance spectroscopy was executed on the synthesized specimens. This electroanalytical technique enables the distinct isolation of electrical responses originating from the bulk grains (dominant within the high-frequency regime), the grain boundaries (predominant within the low-frequency regime), and electrode-material interfaces, each of which uniquely modulates the global capacitive and resistive profile of the system. To systematically evaluate the impact of neodymium (Nd^3+^) substitution on the electrical properties while circumventing analytical redundancy in the subsequent long-form analysis, a preliminary comparative screening was performed across the entire compositional series (*x* = 0, 0.2 and 0.4) and presented in Table 3. This baseline evaluation targeted the evolution of the relative permittivity 
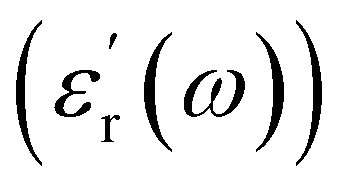
, dissipation factor (tan *δ*), and DC conductivity (*σ*_dc_) at a fixed frequency of 5 × 10^2^ Hz under both ambient (room temperature) and elevated (373 K) thermal states. This frequency lies within the grain-boundary permittivity plateau but is also where conduction-related losses are largest.

**Table 3:** Effect of Nd-substitution (*x* = 0, 0.2, 0.4) on the dielectric properties (
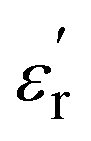
, tan *δ*) and conductivity (*σ*_dc_) of Sm_2−*x*_Nd_*x*_CuO_4_ ceramics at room temperature and 373 K at 5 × 10^2^ Hz

Sm_2−*x*_Nd_*x*_CuO_4_	*x* = 0		*x* = 0.2		*x* = 0.4	
Temperature	Room temperature	373 K	Room temperature	373 K	Room temperature	373 K
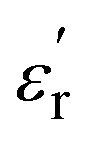	3.10 × 10^3^	3.80 × 10^3^	6.42 × 10^3^	8.80 × 10^3^	1.11 E×10^4^	1.32 × 10^4^
tan *δ*	3.47	9.76	4.40	9.60	7.59	29.33
*σ*_dc_ (S cm^−1^)	2.94 × 10^−6^	1.02 × 10^−5^	7.75 × 10^−6^	2.33 × 10^−5^	2.28 × 10^−5^	1.09 × 10^−4^

The experimental metrics delineate a monotonic correlation between the substitution degree (*x*) and the underlying electrical parameters. At any given temperature, both 
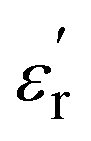
 and *σ*_dc_ scale systematically with increasing Nd content. Specifically, at room temperature 
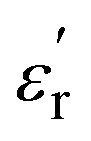
 rises from 3.1 × 10^3^ in the pristine matrix (*x* = 0) to 1.11 × 10^4^ for *x* = 0.4, and from 3.8 × 10^3^ to 1.32 × 10^4^ at 373 K. Concurrently, *σ*_dc_ exhibits a stronger enhancement, of approximately one order of magnitude, rising from 2.94 × 10^−6^ S cm^−1^ to 2.28 × 10^−5^ S cm^−1^ at room temperature, and from 1.02 × 10^−5^ S cm^−1^ to 1.09 × 10^−4^ S cm^−1^ at 373 K. This concomitant enhancement of both capacitive 
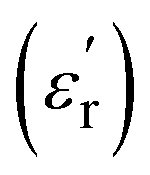
 and conductive (*σ*_dc_) components implies that the substitution of Sm^3+^ by Nd^3+^ induces localized structural alterations and increases the charge carrier density within the Sm_2−*x*_Nd_*x*_CuO_4_ lattice. These induced states subsequently intensify Maxwell–Wagner interfacial polarization and accelerate short-range hopping conduction mechanisms across adjacent potential barriers.

The dissipation factor is, however, high at this frequency: tan *δ* = 3.47–7.59 at room temperature and 9.76–29.33 at 373 K, larger than one for all compositions, so that the investigated ceramics cannot be regarded as low-loss dielectrics under these conditions. Such values are characteristic of the conduction-dominated low-frequency regime of Maxwell–Wagner (IBLC-type) dielectrics, where the loss is governed by the DC leakage through the grain boundaries. At 373 K the disparity widens significantly, particularly *x* = 0.4; this behavior reflects a thermally activated semiconductor-like conduction mechanism, wherein additional thermal energy enables localized charge carriers to overcome trapping potentials, thereby increasing conduction-associated losses. Because this loss is conduction-related, it decreases rapidly with increasing frequency: for *x* = 0.4 at 290 K, tan *δ* falls and remains below 1 between 10 kHz and 1 MHz over the whole investigated temperature range of 260–393 K (inset of Fig. 13a). The high losses observed at 500 Hz should therefore not be generalized to the entire frequency and temperature ranges. Nevertheless, the low-frequency loss remains too high for practical capacitor applications, and Nd substitution should be regarded as a means of enhancing 
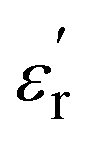
 at the expense of increased leakage. Reducing this loss, for instance through the control of the sintering conditions and of the oxygen stoichiometry, which govern the grain-boundary resistance,^14^ together with further application-specific validation, is required before device integration can be considered.

In summary, the preliminary screening highlights that neodymium substitution effectively increases both the dielectric permittivity and the charge transport, at the expense of a higher conduction-related loss at low frequency. Because the *x* = 0.4 composition exhibits the highest permittivity among the evaluated series and a loss that falls below 1 at higher frequencies, it was selected as a promising candidate for an in-depth electro-physical investigation. Consequently, a comprehensive dielectric analysis of this *x* = 0.4 compound will now be presented, encompassing a detailed evaluation of its complex impedance, electrical conductivity, electrical modulus, and relative permittivity, including the frequency and temperature dependence of the dielectric loss.

#### 3.4.2 Impedance formalism

To systematically decouple the individual electroactive contributions of the bulk grains, grain boundaries, and electrode interfaces, complex impedance spectroscopy was employed. The experimental data for the optimized Sm_1.6_Nd_0.4_CuO_4_ composition is represented using Nyquist formalisms, plotting the imaginary part of the impedance (−*Z*″) against the real part (*Z*′).^47,48^Fig. 6a illustrates the temperature-dependent Nyquist plots for the high-substituted ceramic across a thermal range of 200 K to 393 K. these plots display two distinct, depressed semi-circular arcs: the first at high frequencies, which is attributed to the response of the bulk grains, and the second at low frequencies, corresponding to the grain boundary contribution.^49^

**Fig. 6:**
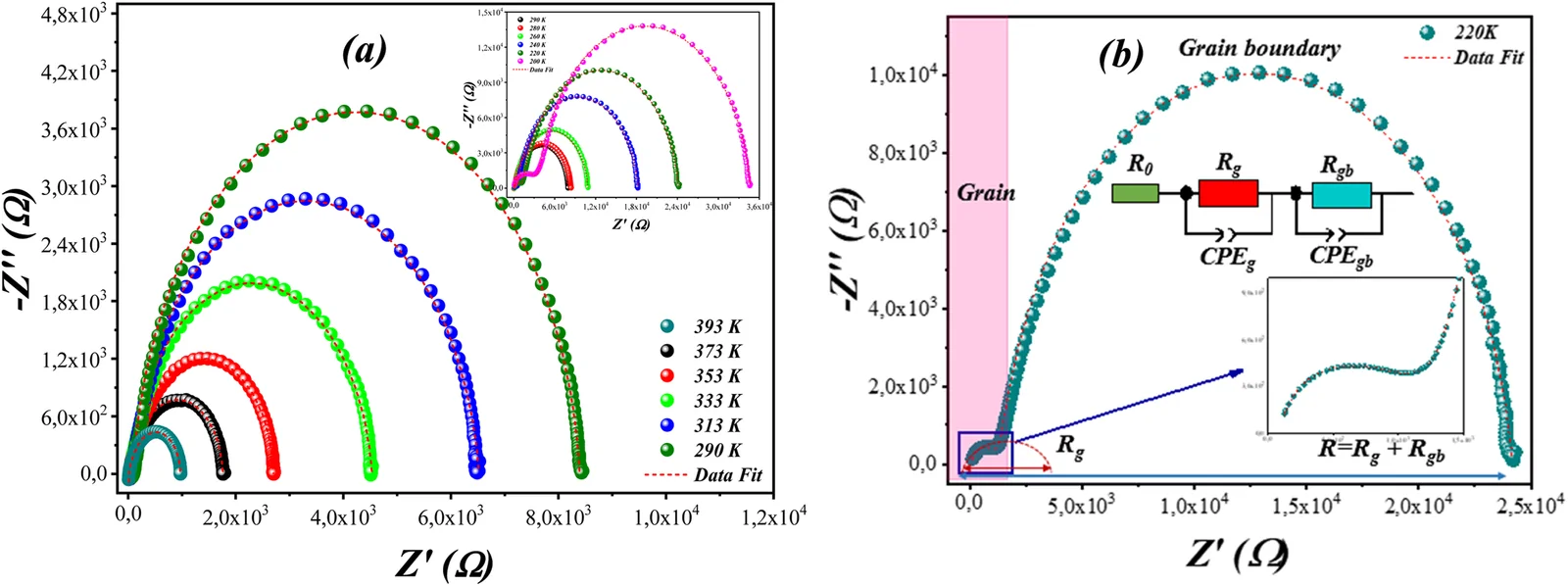
Nyquist plots of sample Sm_1.6_Nd_0.4_CuO_4_ (a) for low and high temperatures and (b) at temperature 220 K, over the wide frequency range [10–13 × 10^7^ Hz], as well as best fits (solid lines) corresponding to equivalent circuits. The zoomed-in inset illustrates the grain response.

As the thermal energy in the system increases, the apex of the arcs shifts toward higher frequencies, and the overall diameter of the semicircles, which correlates directly with the total electrical resistance of the material, decreases drastically. This marked reduction in impedance with increasing temperature is a classic manifestation of a Negative Temperature Coefficient of Resistance (NTCR). This behavior confirms the thermally activated semiconducting nature of the synthesized cuprates, which is highly consistent with trends observed in other high-permittivity complex perovskite oxides^50–52^ as well as in related copper oxide system like SCO.^53^ This semiconducting character, which mainly concerns the grains, is not in contradiction with a high dielectric constant: in ceramics of the internal barrier layer capacitor (IBLC) type, the giant permittivity is of extrinsic (Maxwell–Wagner) origin and requires semiconducting grains separated by thin insulating grain boundaries.^50,54^

To extract quantitative physical parameters from these macroscopic measurements, the impedance spectra were modelled and fitted using an equivalent circuit.^55,56^ Because real polycrystalline materials inherently possess microstructural heterogeneities, ideal Debye relaxation models consisting of pure parallel Resistor-Capacitor circuits are insufficient. Fitting was therefore carried out using *Z*-View software. Fig. 6b presents a representative Nyquist profile captured at 220 K, where the distinct relaxation regimes are clearly resolvable. This spectrum is perfectly fitted using the equivalent circuit model *R*_0_ + (*R*_g_//CPE_g_) + (*R*_gb_//CPE_gb_) depicted in the inset, where *R*_0_, *R*_g_ and *R*_gb_ represent the contact/system resistance, bulk grain resistance and grain boundary resistance, respectively.^48,49^ The implementation of Constant Phase Elements (CPE) rather than ideal discrete capacitors is mathematically necessary to account for the “depressed” nature of the semicircles (where the center of the arc lies below the real *Z*′ axis). This depression indicates a distribution of relaxation times within the lattice, driven by non-ideal capacitor behavior, localized lattice strain, and atomic-scale defect gradients. All physical parameters deduced from this equivalent circuit fitting are comprehensively catalogued in Table 4.

**Table 4:** Fitted parameter values; resistances (*R*_g_, *R*_gb_) and capacitances (*C*_g_, *C*_gb_) associated with grains and grain boundaries obtained from simulation of Nyquist plots using the equivalent circuit model 2R-CPE for sample Sm_1.6_Nd_0.4_CuO_4_ at different temperature

*T* (K)	*R*_g_ (kΩ)	*R*_gb_ (kΩ)	*C*_g_ (F)	*C*_gb_ (F)
200	3.67	3.02 × 10^1^	3.97 × 10^−11^	3.85 × 10^−9^
220	1.81	2.01 × 10^1^	4.68 × 10^−11^	3.94 × 10^−9^
240	1.24	1.46 × 10^1^	5.01 × 10^−11^	4.17 × 10^−9^
260	8.88 × 10^−1^	1.05 × 10^1^	5.86 × 10^−11^	4.37 × 10^−9^
280	6.33 × 10^−1^	8.51	6.28 × 10^−11^	4.61 × 10^−9^
290	5.38 × 10^−1^	7.27	7.02 × 10^−11^	4.93 × 10^−9^
313	3.68 × 10^−1^	5.81	7.36 × 10^−11^	5.11 × 10^−9^
333	1.34 × 10^−1^	3.97	8.28 × 10^−11^	5.61 × 10^−9^
353	1.90 × 10^−2^	2.73	9.11 × 10^−11^	5.87 × 10^−9^
373	1.20 × 10^−2^	1.78	9.82 × 10^−11^	6.01 × 10^−9^
393	0.98 × 10^−2^	0.96	9.97 × 10^−11^	7.47 × 10^−9^

The dynamic electrical response of the optimized Sm_1.6_Nd_0.4_CuO_4_ ceramic is illustrated by the frequency dependence of the real impedance (*Z*′), presented in Fig. 7a (200–393 K). in the low-frequency regime, *Z*′ establishes a plateau corresponding to the total DC resistance, which decreases monotonically as temperature rises. This thermal reduction confirms a fundamental Negative Temperature Coefficient of Resistance (NTCR) typical of semiconductors.^57^ Beyond a characteristic frequency, *Z*′ exhibits a gradual roll-off indicating dielectric relaxation, eventually merging into a temperature-independent continuum near 10^6^ Hz due to the release of space charge polarization.^58^

**Fig. 7:**
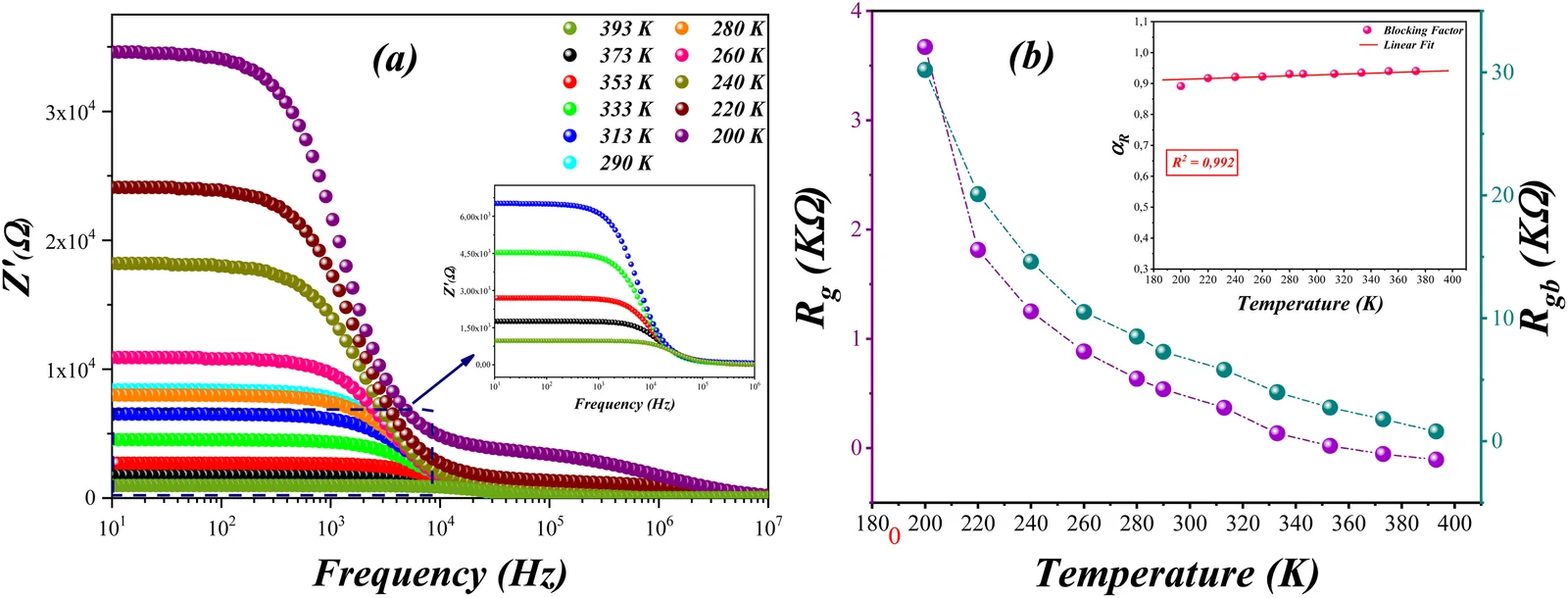
(a) Frequency-dependent dispersion of the real impedance (*Z*′) as a function of temperature (200–393 K), (b) extracted bulk grain (*R*_g_) and grain boundary (*R*_gb_) resistances plotted against temperature; the inset depicts the temperature-dependent modulation of the grain boundary blocking factor.

To pinpoint the microstructural origins of this conduction, the bulk grain (*R*_g_) and grain boundary (*R*_gb_) resistances were extracted from equivalent circuit modelling and plotted against temperature (Fig. 7b). Both components exhibit an exponential thermal decay, confirming a thermally activated conduction mechanism. However, *R*_gb_ remains substantially larger than *R*_g_ across the entire thermal range. To quantify this interfacial dominance, the grain boundary blocking factor (*α*_R_) was evaluated:2
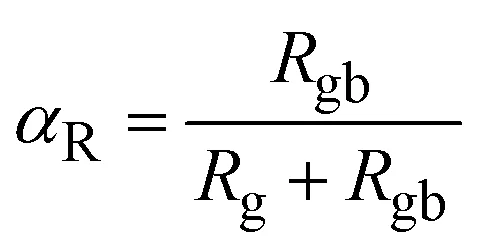


As shown in the inset of Fig. 7b, *α*_R_ remains exceptionally high (∼0.9) at all temperatures, showing that about 90% of the total resistance is located at the grain boundaries, which dictate the global electrical transport. This pronounced bottleneck effect is typically driven by microstructural factors such as intergranular variations in oxygen stoichiometry, charge carrier trapping in pores, crystallographic disorder at interfaces, or secondary phase segregation.^59,60^ Consequently, while hopping conduction occurs freely within the bulk grains, the grain boundaries act as the primary macroscopic potential barriers. This configuration of semiconducting grains separated by resistive grain boundaries is precisely the IBLC architecture responsible for the giant permittivity of the material.

The dynamic relaxation behavior of the optimized Sm_1.6_Nd_0.4_CuO_4_ ceramic is further analysed through the frequency dependence of the imaginary part of the impedance (*Z*″) and its normalized representation (*Z*″/
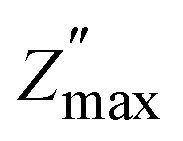
), as presented in Fig. 8a. The isothermal spectra exhibit distinct loss peaks corresponding to a characteristic relaxation frequency (*f*_r_). As the temperature increases, these peaks systematically shift toward higher frequency regimes accompanied by a reduction in peak amplitude, confirming the presence of a thermally activated dielectric relaxation phenomenon.^60^

**Fig. 8:**
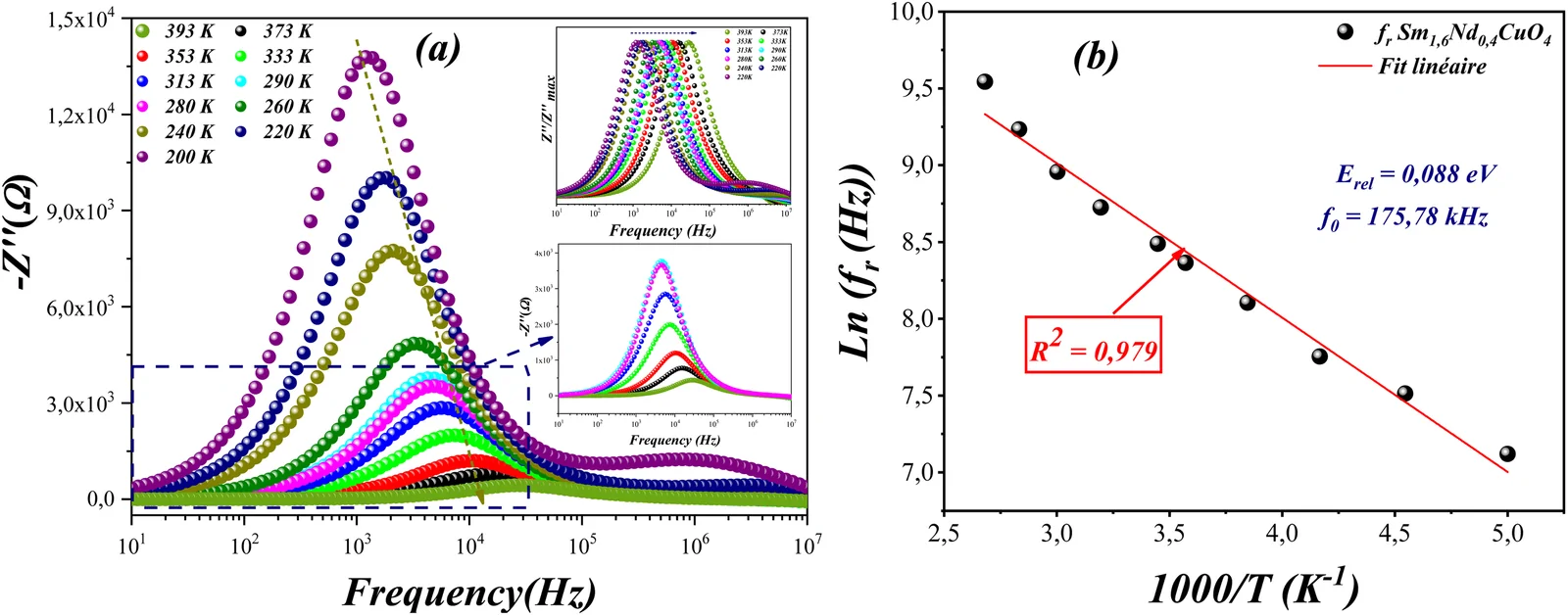
(a) Frequency dependence of the imaginary part of impedance (*Z*″) and its normalized representation (
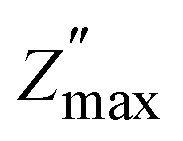
/*Z*″) at various temperatures for the Sm_1.6_Nd_0.4_CuO_4_ ceramic. (b) Arrhenius plot illustrating the temperature dependence of the relaxation frequency (*f*_r_) *vs.* 1000/T.

To quantify the energy barrier governing this short-range mobility, the temperature dependence of the extracted relaxation frequency (*f*_r_) is plotted as ln(*f*_r_) *versus* 1000/*T* in Fig. 8b. This linear correlation is accurately modelled using the empirical Arrhenius relationship:3
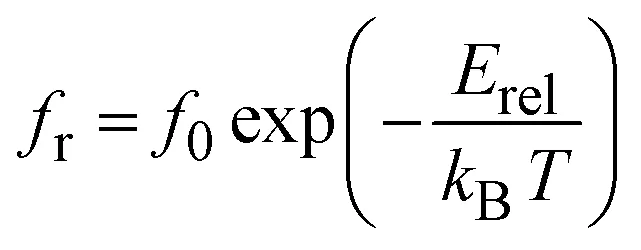
where *f*_0_ is the pre-exponential factor (representing the intrinsic attempt frequency at infinite temperature), *E*_rel_ is the activation energy required for the dielectric relaxation process, *k*_B_ is the Boltzmann constant, and *T* is the absolute temperature. Linear regression of the experimental data yields an activation energy *E*_rel_ = 0.088 eV and a pre-exponential factor *f*_0_ = 175.75 kHz, indicative of localized hopping mechanisms within the highly resistive grain boundaries.

#### 3.4.3 Electrical conductivity and charge transport kinetic

The frequency-dependent alternating current (AC) electrical conductivity (*σ*) of the Sm_1.6_Nd_0.4_CuO_4_ ceramic was systematically evaluated across a broad thermal matrix to elucidate the underlying charge transport kinetics. The total conductivity was deduced using the complex impedance data in conjunction with the geometric dimensions of the sample pellet according to the relation:4
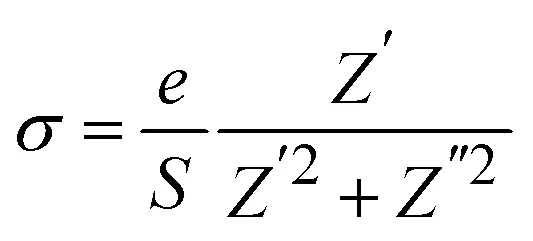
where *e* and *S* represent the thickness and cross-sectional area of the pellet, respectively. The resulting conductivity spectra, plotted across various temperatures in Fig. 9a, exhibit a universal dielectric dispersion characteristic of complex perovskite oxides. Each spectrum presents a distinct low-frequency plateau, representing the frequency-independent direct current (DC) conductivity (*σ*_dc_), followed by a high-frequency dispersive regime. This dynamic behavior is effectively modeled by Jonscher's universal law:^61^

5 *σ*(*ω*) = *σ*_dc_ + *σ*_ac_ = *σ*_dc_(*T*) + *Aω*^*s*^

where *A* is a temperature-dependent pre-exponential factor, *ω* is the angular frequency, and “*s*” is the dimensionless frequency exponent (0 ≤ *s* ≤ 1 or slightly higher). As demonstrated in Fig. 9b, the exponent *s* is directly extracted from the linear fit of the high-frequency dispersive region, serving as a critical diagnostic tool for identifying the dominant localized conduction mechanism.

**Fig. 9:**
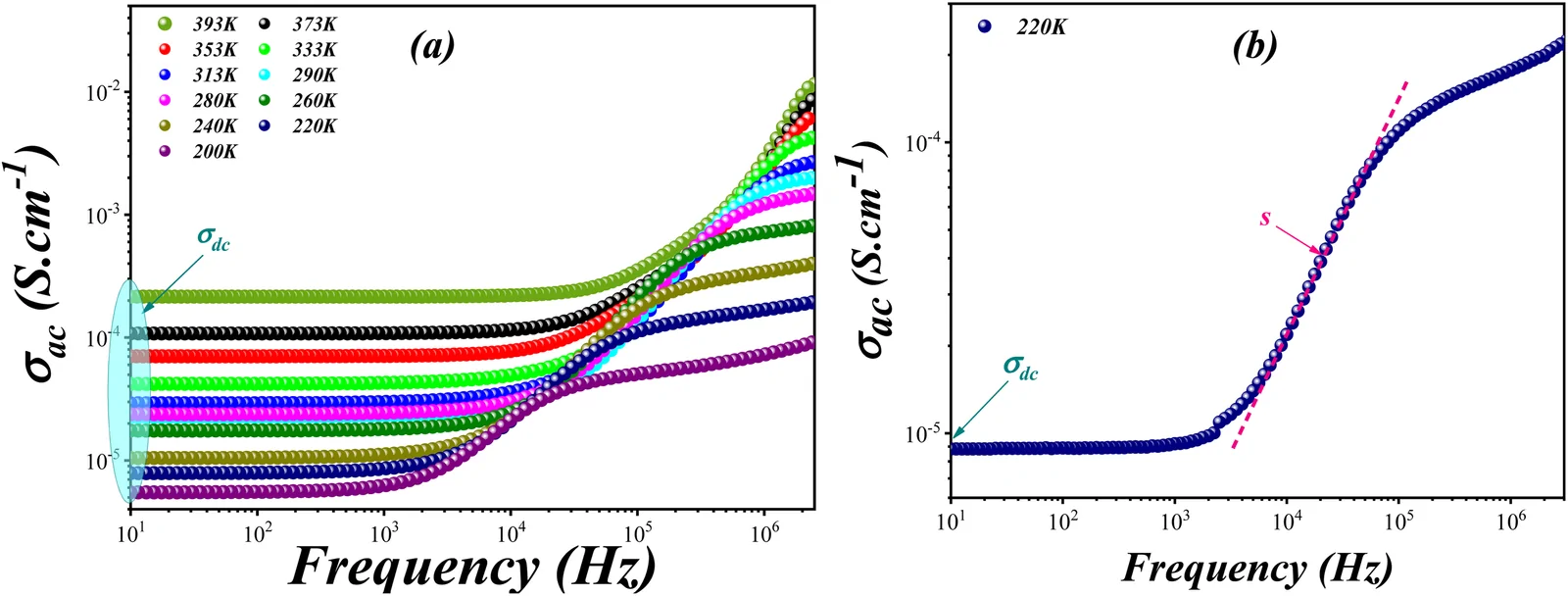
(a) Variation of *σ*_ac_ with frequency for various temperatures of Sm_1.6_Nd_0.4_CuO_4_ and (b) identification of the exponent (*S*) based on Jonscher's universal power law.

To discern the precise quantum mechanical nature of the localized transport processes, the temperature dependence of the extracted frequency exponent (*s*) was carefully evaluated. As illustrated in Fig. 10a, the experimental values of *s* exhibit a monotonic, continuous increase with rising temperature. This positive thermal correlation is a classic fingerprint of the Non-Overlapping Small Polaron Tunneling (NSPT) mechanism.^62^ Under the NSPT framework, small polarons are formed when localized charge carriers induce structural distortions in the surrounding lattice.

**Fig. 10:**
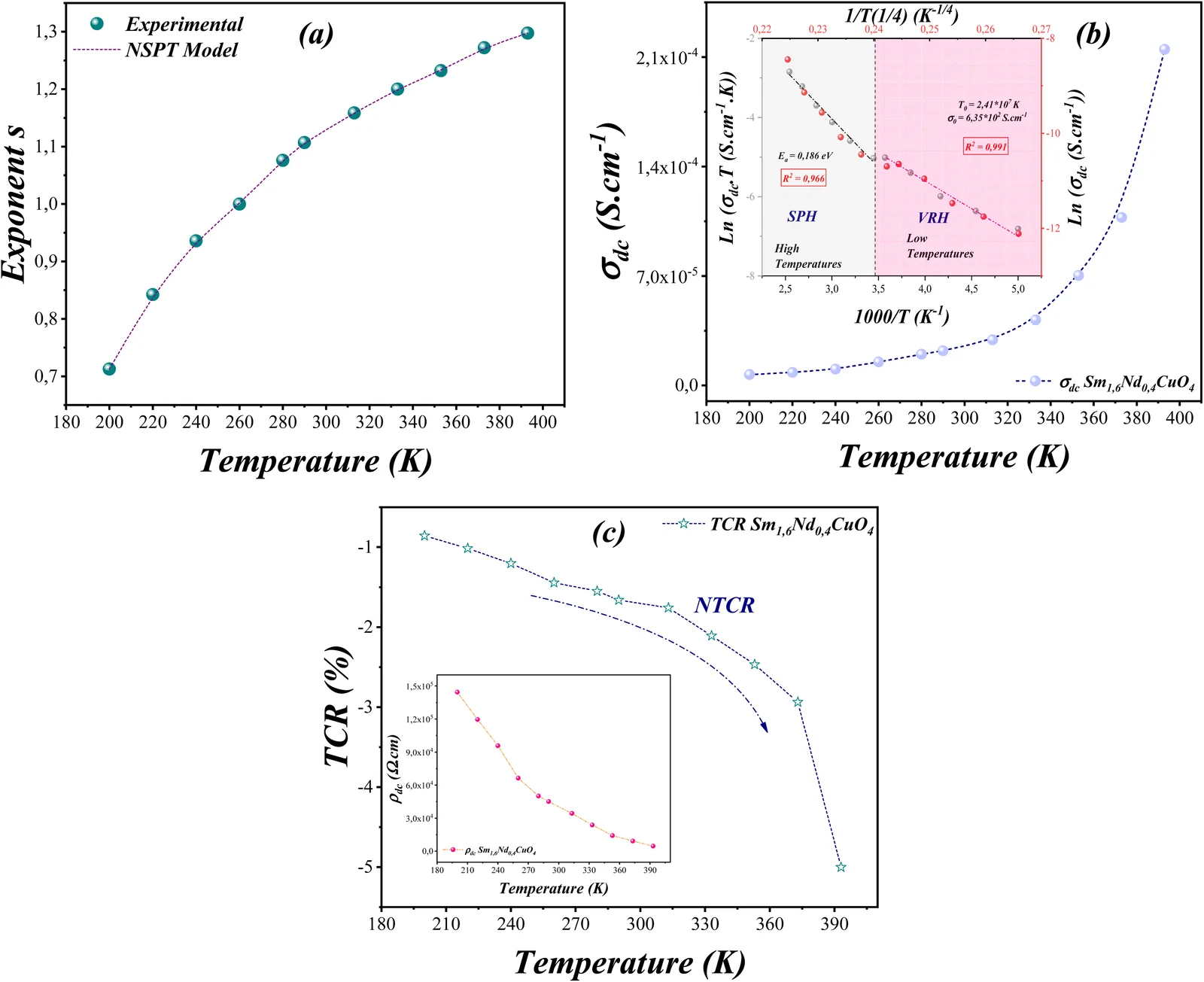
(a) Temperature-dependent behavior of exponent *S*. (b) *σ*_dc_ as function of temperature (200−393 K); inset shows Arrhenius plot of ln(*σ*_dc_ × *T*) *vs.* 1000/*T* (bottom and left axes) with the SPH fit, and ln(*σ*_dc_) *vs. T*^−1/4^ (top and right axes) with the VRH fit. (c)Thermal variation of TCR for Sm_1.6_Nd_0.4_CuO_4_ ceramic.

The long-range, stationary charge transport kinetics were further investigated *via* the thermal evolution of the DC conductivity (*σ*_dc_) as shown in Fig. 10b, *σ*_dc_ undergoes an exponential enhancement with increasing temperature, unequivocally confirming the semiconducting nature of the cuprate ceramic. Note that *σ*_dc_ is deduced from the total resistance, which is dominated by the grain boundaries (*α*_R_ ≈ 0.9). To differentiate the governing transport models across distinct thermal regimes, the data were analyzed using the Mott–Davis framework. The corresponding Arrhenius plot (inset of Fig. 10b), ln(*σ*_dc_ × *T*) *vs.* 1000/*T* and ln(*σ*_dc_) *vs. T*^−1/4^, reveal two distinct linear regions separated by a noticeable crossover point near 290 K, indicating a temperature-dependent transition in the dominant charge transport mechanism:

• Low-temperature regime (*T* ≤ 290 K): the transport is best described by the Variable Range Hopping (VRH) model.^63,64^ In this low thermal energy state, electrons hop over varying distances to find energetically favorable localized defect states. In this model, the DC conductivity follows:6
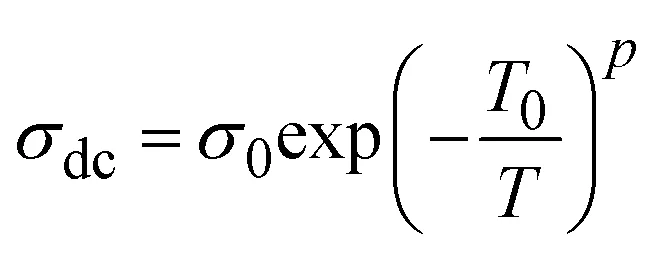
where *p* = 1/(*d* + 1) depends on the dimensionality *d* of the hopping process 
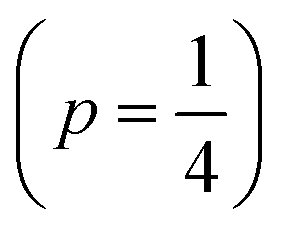
 for *d* = 3, *σ*_0_ is a pre-exponential factor, and *T*_0_ = *B*_0_/*k*_B_*N*(*E*_F_)*ξ*^*d*^)) is the characteristic Mott temperature, with *N*(*E*_F_) the density of localized states at the Fermi level, *ξ* the localization length and *B*_0_ a numerical constant.

• High-temperature regime (*T* ≥ 290 K): the transport shifts to the Small Polaron Hopping (SPH) model, plotted as ln(*σ*_dc_·*T*) *versus* 1000/*T*, according to the classical Arrhenius expression:7
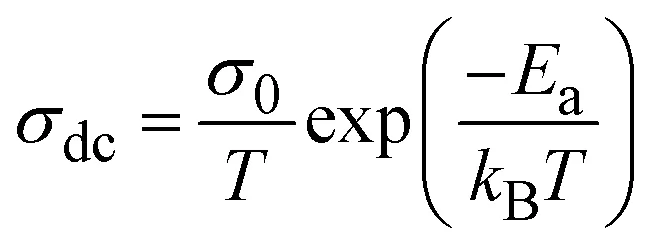
With *σ*_0_ is defined as a pre-exponential factor, *k*_B_ presents Boltzmann's constant and *E*_a_ will be the activation energy related to the height of the potential energy barrier limiting charge carrier motion.

The linear evolution of ln(*σ*_dc_) *vs. T*^−1/4^ for *T* ≤ 290 K indicates a three-dimensional VRH mechanism and gives *T*_0_ = 2.41 × 10^7^ K and *σ*_0_ = 6.35 × 10^2^ S cm^−1^ (*R*^2^ = 0.991). The value of *T*_0_ is of the same order of magnitude as that reported for the parent compound (1.65 × 10^7^ K) and for other oxides.^65^

Linear regression in this regime yields an activation energy of *E*_a_ ≈ 0.186 eV. This value is slightly lower than that of the parent compound (0.197 eV^14^), consistent with the increase of *σ*_dc_ and of the permittivity upon Nd substitution and lies in the same range as the values found in our other cuprate ceramics exhibiting giant permittivity (0.248 eV in GdCa_2_Cu_3_O_*δ*_ ref. 13 and 0.23–0.26 eV in YCa_2_Cu_3_O_*δ*_).^41^ Such a relatively low activation energy is characteristic of semiconducting grains, whose carriers are thermally activated by hopping among Cu^2+^/Cu^3+^ sites and by the ionisation of oxygen vacancies;
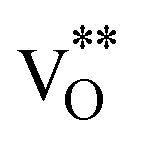
^66,67^ it does not imply that the grain boundaries are conductive. On the contrary, the semiconducting behavior of the grains is what the IBLC mechanism requires: it provides the mobile carriers that accumulate at the insulating grain boundaries under the AC field and give rise to the interfacial (Maxwell–Wagner) polarization responsible for the giant permittivity.^68^ During high-temperature processing or sintering under low oxygen partial pressure, the volatilization of oxygen generates these vacancies alongside weakly bound electrons according to the following reactions:8
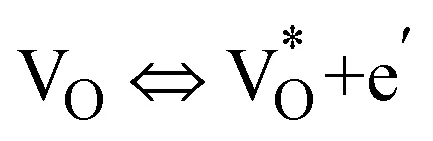
9
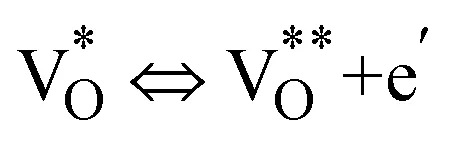


These thermally activated electrons are captured by mixed-valence copper ions (Cu^3+^/Cu^2+^), facilitating small polaron hopping and driving the macroscopic electrical conductivity at elevated temperature.^67^

To systematically assess the thermal sensitivity of the Sm_1.6_Nd_0.4_CuO_4_ ceramic for potential integration into uncooled infrared bolometers and thermal sensing technologies, the Temperature Coefficient of Resistance (TCR) was quantified. The TCR defines the relative change in electrical resistivity (*ρ*_dc_ = 1/*σ*_dc_) as function of temperature and is mathematically expressed as 
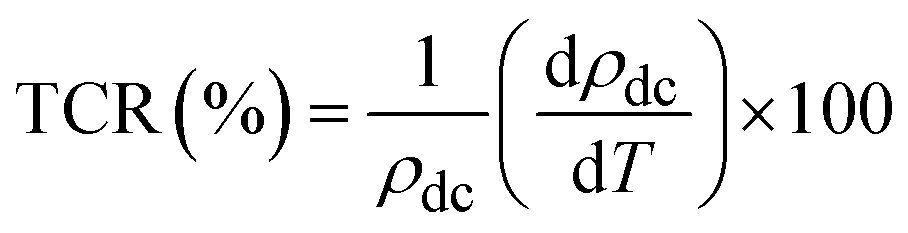
.^24^ The calculated TCR is plotted as function of temperature in Fig. 10c. in strict alignment with the previously established transport behavior, the TCR values remain entirely negative across the evaluated matrix, confirming the Negative Temperature Coefficient of Resistance.

The experimental TCR curve exhibits a pronounced thermal evolution. At ambient conditions, the material demonstrates a moderate baseline sensitivity. However, as the thermal energy increases and the system fully transitions into the SPH regime, the TCR magnitude undergoes a sharp, near-linear enhancement. It reaches a highly pronounced maximum sensitivity peak of approximately −5% near 390 K. this maximum TCR magnitude represents a highly competitive and superior value compared to standard commercial uncooled bolometer material (such as amorphous silicon or vanadium oxide, which typically yield maximum TCR values ranging from −2.1% to −4%,^69,70^ underscoring the industrial potential of this heavily substituted cuprate system.

#### 3.4.4 Electric modulus analysis

The complex electric modulus formalism is a powerful tool for investigating conductivity and relaxation behaviors in oxide materials, as it effectively suppresses electrode polarization effects and highlights bulk/grain boundary responses. The frequency-dependent complex electrical modulus M*(*ω*) is defined as:

10 M*(*ω*) = *M*′(*ω*) + *jM*″(*ω*) = *jωC*_0_Z*(*ω*)

where *C*_0_ is the geometric capacitance of the vacuum, *ω*is the angular frequency, and *M*′ and *M*″ represent the real and the imaginary parts of the electrical modulus, respectively.

Fig. 11a displays the frequency dependence of the real part of the modulus *M*′ across a wide temperature range. At low frequencies, *M*′ exhibits extremely low values, which indicates a negligible contribution from electrode and/or ionic polarization.^71^ As the frequency increases, *M*′ demonstrates a continuous dispersion, gradually increasing before showing a tendency to saturate at the highest frequencies. This high-frequency saturation corresponds to the confinement of charge carriers within their potential energy wells, rendering them insensitive to the rapidly alternating applied electrical field.^72,73^

**Fig. 11:**
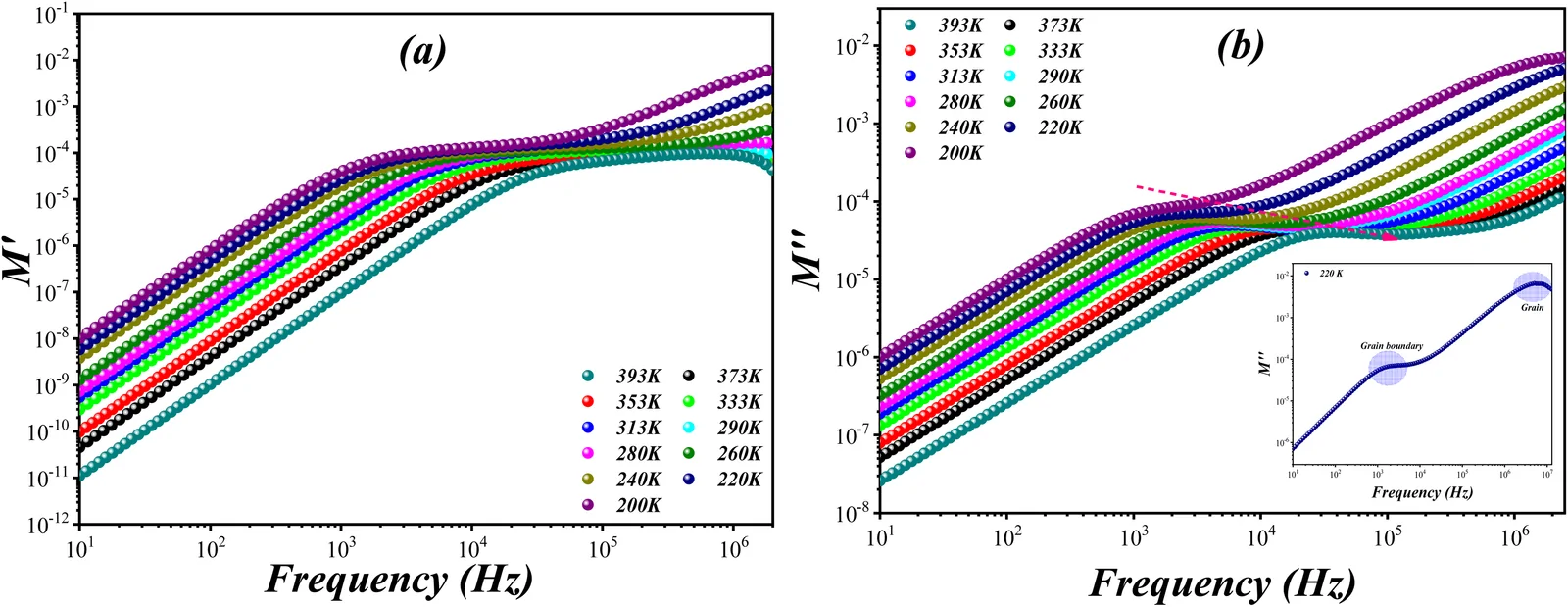
Frequency dependence of the electric modulus at selected temperature (a) real part *M*′ and (b) imaginary part *M*″, over the temperature range from 200 K to 393 K.

The variation of the imaginary part *M*″ with frequency is presented in Fig. 11b. In contrast to the *Z*″ spectra, which exhibit well-defined loss peaks, the *M*″ spectra display broad, overlapping features: an intermediate-frequency shoulder assigned to grain boundaries and a steep high-frequency rise toward the grain interior peak near the upper measurement limit. As temperature increases, both features shift systematically to higher frequencies, consistent with the thermally activated process observed in the impedance formalism. The broad, asymmetric shapes reflect a distribution of relaxation times (non-Debye behavior^74^), aligning with the depressed arcs and constant phase elements (CPE) used in the equivalent circuit.

Notably, as highlighted in the inset of Fig. 11b for the 220 K spectrum, the modulus representation effectively deconvolutes the microstructural contributions across approximately three frequency decades The grain-boundary contribution appears as a shoulder rather than as a peak because the height of the *M*″ maximum is inversely proportional to the capacitance (
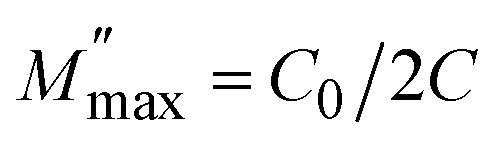
, with *C*_0_ = *ε*_0_*S*/*e*). Because *C*_gb_ is one to two orders of magnitude larger than *g* (Table 4), the grain-boundary maximum is correspondingly smaller and superimposes on the low-frequency flank of the dominant grain response. Because these features shift continuously with temperature, the assignment established at 220 K applies across the entire temperature range.

To further elucidate the nature of charge carrier mobility, the imaginary modulus (*M*″) and the imaginary impedance (−*Z*″) are plotted together for selected temperatures in Fig. 12 According to established literature, a significant frequency mismatch between the *M*″ and *Z*″ peaks (*f*_max_(*M*″) ≠ *f*_max_(*Z*″)) is indicative of localized, short-range charge movement.^75–77^ However, for the Sm_1.6_Nd_0.4_CuO_4_ ceramic at both 290 K (Fig. 12a) and 393 K (Fig. 12b), the characteristic peaks of *Z*″ and *M*″ perfectly coincide at the same frequency. This precise alignment (*f*_max_(*M*″) ≈ *f*_max_(*Z*″)) conclusively demonstrates that the primary relaxation mechanism in this thermal regime is delocalized, driven by the long-range translational mobility of charge carriers successfully hoping between adjacent sites.

**Fig. 12:**
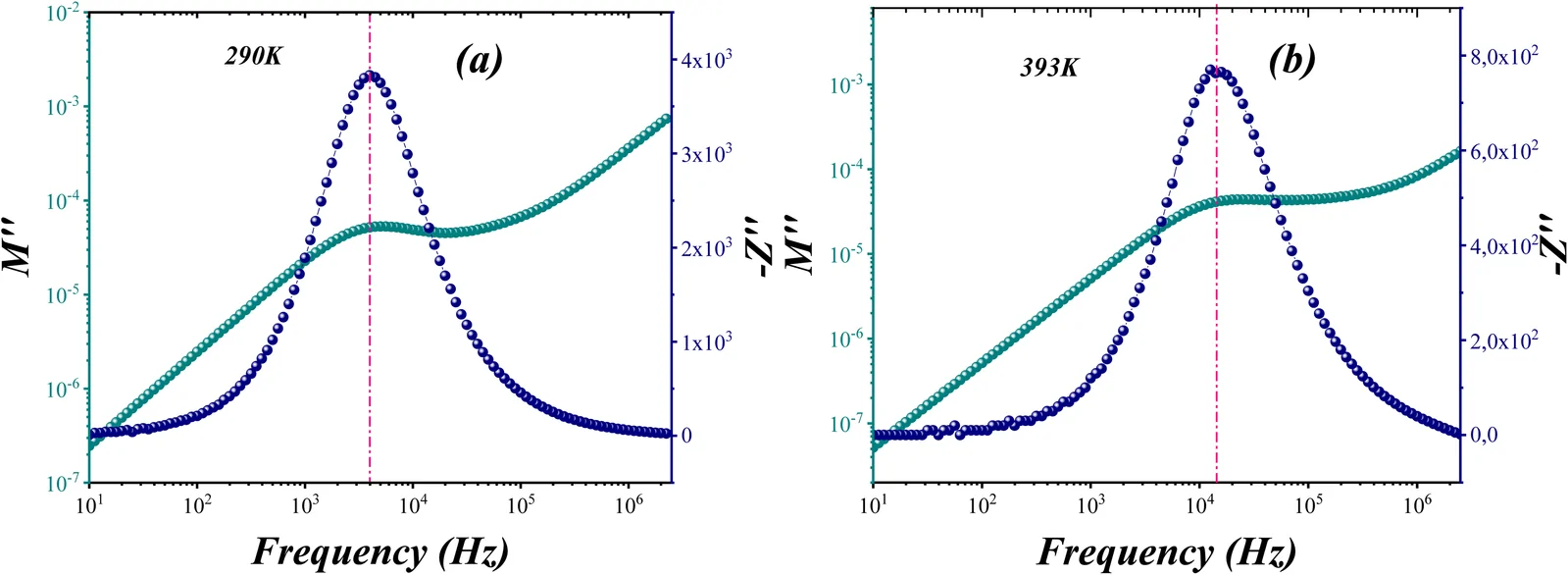
The imaginary part of impedance (*Z*″) and modulus (*M*″) *versus* frequencies at selected temperatures ((a) 290 and (b) 393 K).

#### 3.4.5 Complex dielectric permittivity and relaxation kinetics

To gain deeper insights into the capacitive charge storage capacity, polarization mechanisms, and interfacial microstructural properties of the prepared solid-state ceramics, the frequency-dependent complex dielectric permittivity formalism (ε* = *ε*′ + *iε*″) was systematically investigated. The real part of the dielectric permittivity (*ε*′), which quantifies the material's ability to store electrical energy under an applied alternating current (AC) field, and the imaginary part (*ε*″), which reflects the energy dissipated as thermal losses, were extracted from the complex impedance spectra. These parameters, along with the dielectric loss (tan *δ*), are calculated using standard geometric and electrostatic relationships:^14,78^11
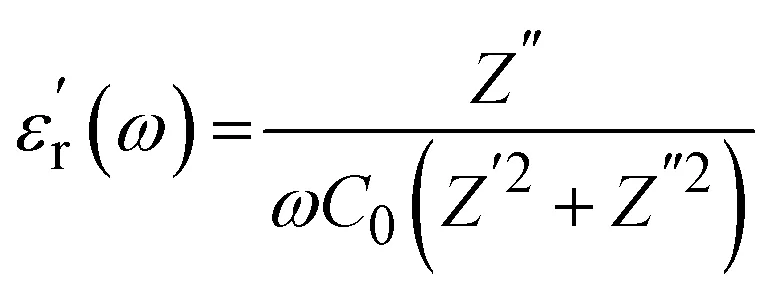
12
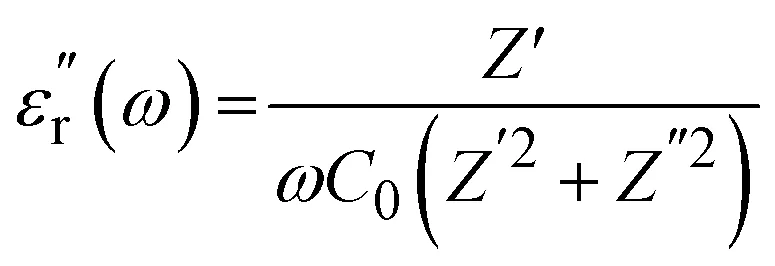
13
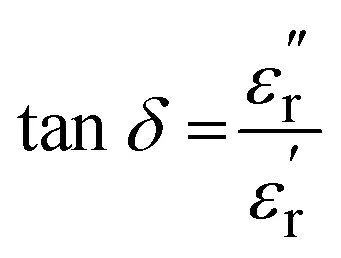
where *ω* = 2π*f* is the angular frequency of the applied field, and *C*_0_ = *ε*_0_*S*/*e* represents the vacuum capacitance of the empty cell configuration. Here, *ε*_0_ is the free-space permittivity, *S* is the cross-sectional area of the silver-lacquered pellet electrodes, and *e* the thickness of the sample).^79,80^

The evolution of the dielectric constant 
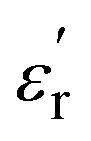
 and the dielectric loss tan *δ* as a function of frequency and temperature for the Sm_1.6_Nd_0.4_CuO_4_ ceramic is illustrated in Fig. 13a. Above room temperature, the compound exhibits a distinct step-like dielectric dispersion characterized by two primary plateaus:

**Fig. 13:**
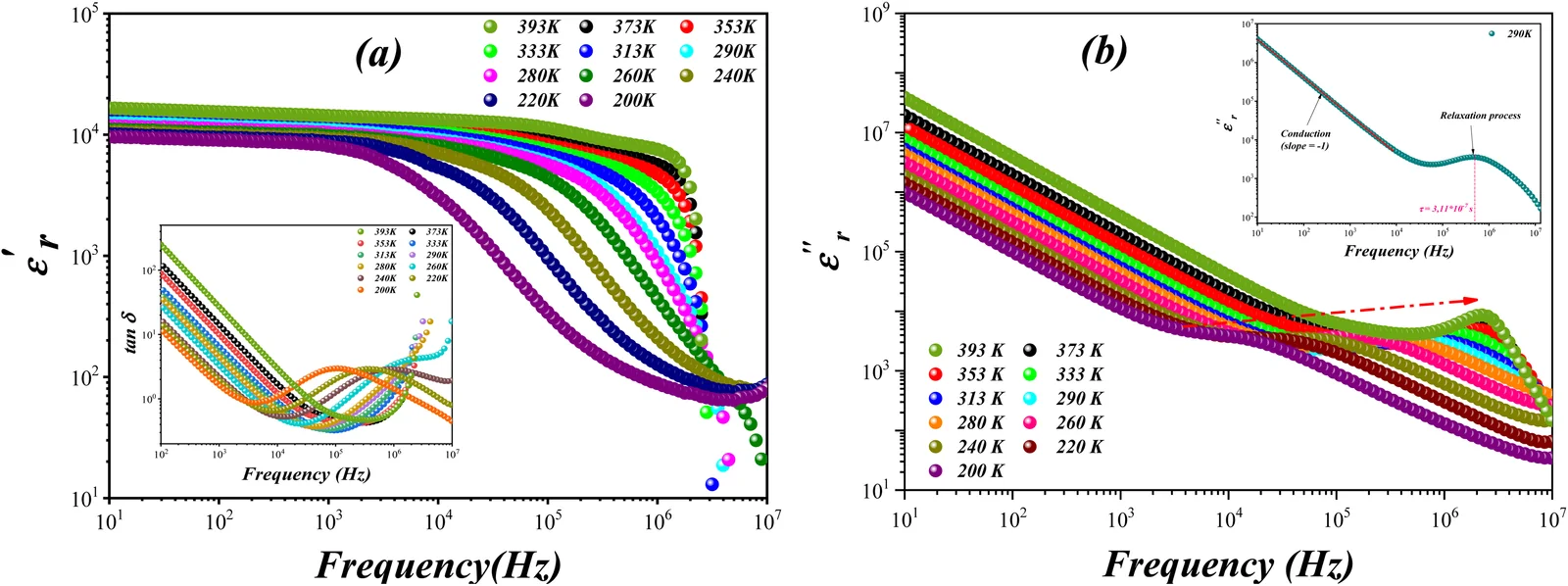
Frequency dependence of dielectric properties for the Sm_1.6_Nd_0.4_CuO_4_ ceramic at various temperatures: (a) real part of the relative permittivity 
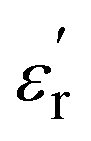
, with the inset displaying the corresponding dielectric loss (tan *δ*); (b) imaginary part of the relative permittivity relative permittivity 
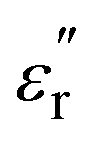
, with the inset detailing the 290 K spectrum to highlight the low-frequency conduction region and the high-frequency relaxation peak.

• Low-to-intermediate frequencies (10 Hz to 10^4^ Hz): the material demonstrates a colossal permittivity 
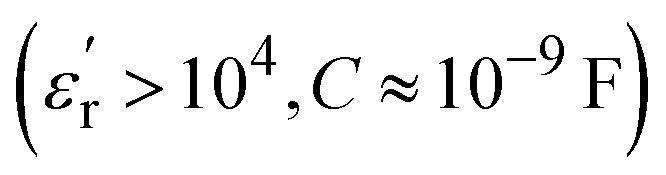
. This massive energy storage capacity is primarily attributed to the Internal Barrier Layer Capacitor (IBLC) effect at the grain boundaries, which favours the polarization of space charges and dipoles at defect sites, such as oxygen vacancies or microstructural porosity.

• High frequencies (10^6^ Hz to 10^7^ Hz): the permittivity drops dramatically to a lower plateau (
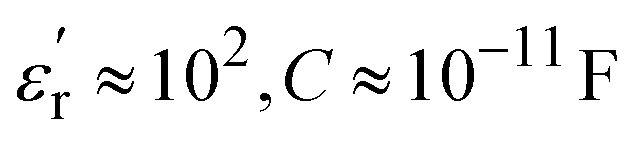
), representing the intrinsic bulk response of the semiconducting grains. This behavior closely mirrors previous observations in related cuprate systems.^14,24^

This colossal dielectric behavior exhibits a classic Debye-type relaxation. According to the Maxwell–Wagner polarization model,^81,82^ this phenomenon is linked to the insulating barriers formed between the semiconducting grains.^83,84^ In the low-frequency regime (*ωτ* ≪ 1, with *τ* presents the relaxation time), dipoles can easily track the alternating electric field, keeping 
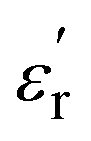
 stable and minimizing the dielectric loss to a low value. As the frequency approaches the relaxation condition *ωτ* = 1), a transition occurs, marked by a peak in tan *δ* (inset of Fig. 13a). At high frequencies (*ωτ* ≫ 1), the relaxation polarization becomes too sluggish to follow the rapid field variations, causing a sharp decline in variations in 
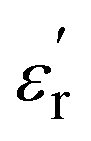
.

The corresponding imaginary permittivity 
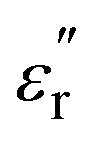
, shown in Fig. 13b, reveals high values at low frequencies that decrease linearly with a slope of approximately −1. This confirms that low-frequency losses are dominated by long-range direct current (DC) electrical conduction and charge accumulation at the electrode-material interfaces. A distinct relaxation peak is visible across all temperatures, shifting to higher frequencies as thermal energy increases, which is indicative of thermally activated dipole moments.^85^

#### 3.4.6 Thermal stability and capacitor applications

The practical viability of colossal permittivity ceramics in commercial microelectronics hinges entirely on their thermal stability and loss profile. Fig. 14 illustrates the temperature dependence of (a) 
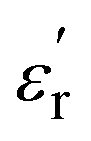
 and (b) tan *δ* at selected intermediate frequencies (10^2^ Hz to 10^3^ Hz). The Sm_1.6_Nd_0.4_CuO_4_ ceramic demonstrates remarkably stable permittivity values alongside low dielectric losses across a broad thermal operational window.

**Fig. 14:**
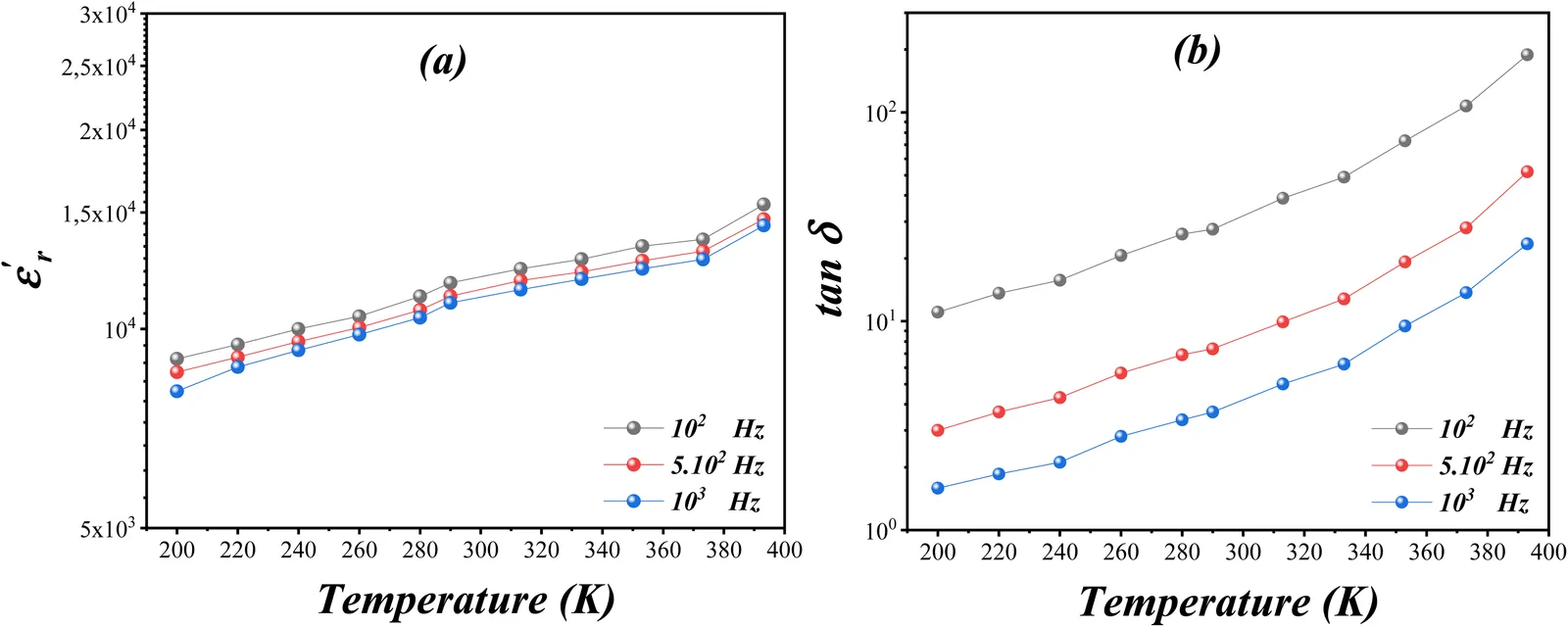
Temperature dependence of the dielectric properties for Sm_1.6_Nd_0.4_CuO_4_ ceramic at selected frequencies: (a) real part of the relative permittivity 
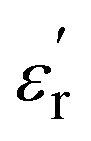
, and (b) dielectric loss (tan *δ*).

To definitively evaluate this material for industrial capacitor applications, the temperature coefficient of the dielectric constant (
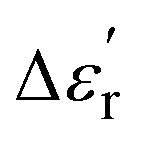
) was calculated relative to room temperature (*T*_amb_) using the relation:^86^14
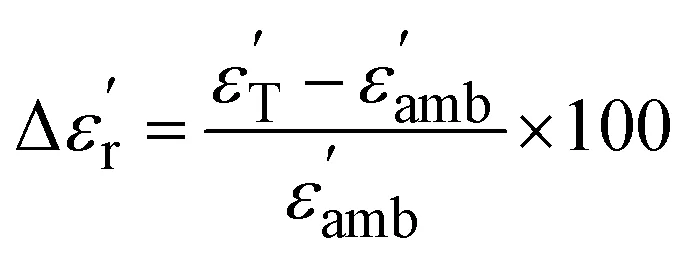


The results yield the most significant finding of this study, as presented in Fig. 15. Standard Electronic Industries Alliance (EIA) RS-198 specifications dictate rigorous tolerances for commercial capacitors.^87^ As shown in Fig. 15a, the pristine Sm_2_CuO_4_ parent compound meets the X5R classification, maintaining a permittivity variation within ±15% over a temperature range of −55 to 85 °C. however, as strikingly demonstrated in Fig. 15b, the stoichiometric substitution of Neodymium structurally optimizes the lattice, restricting the relative variation 
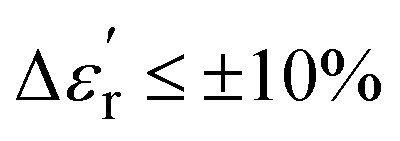
 over an extended temperature range from −55 °C to 105 °C.

**Fig. 15:**
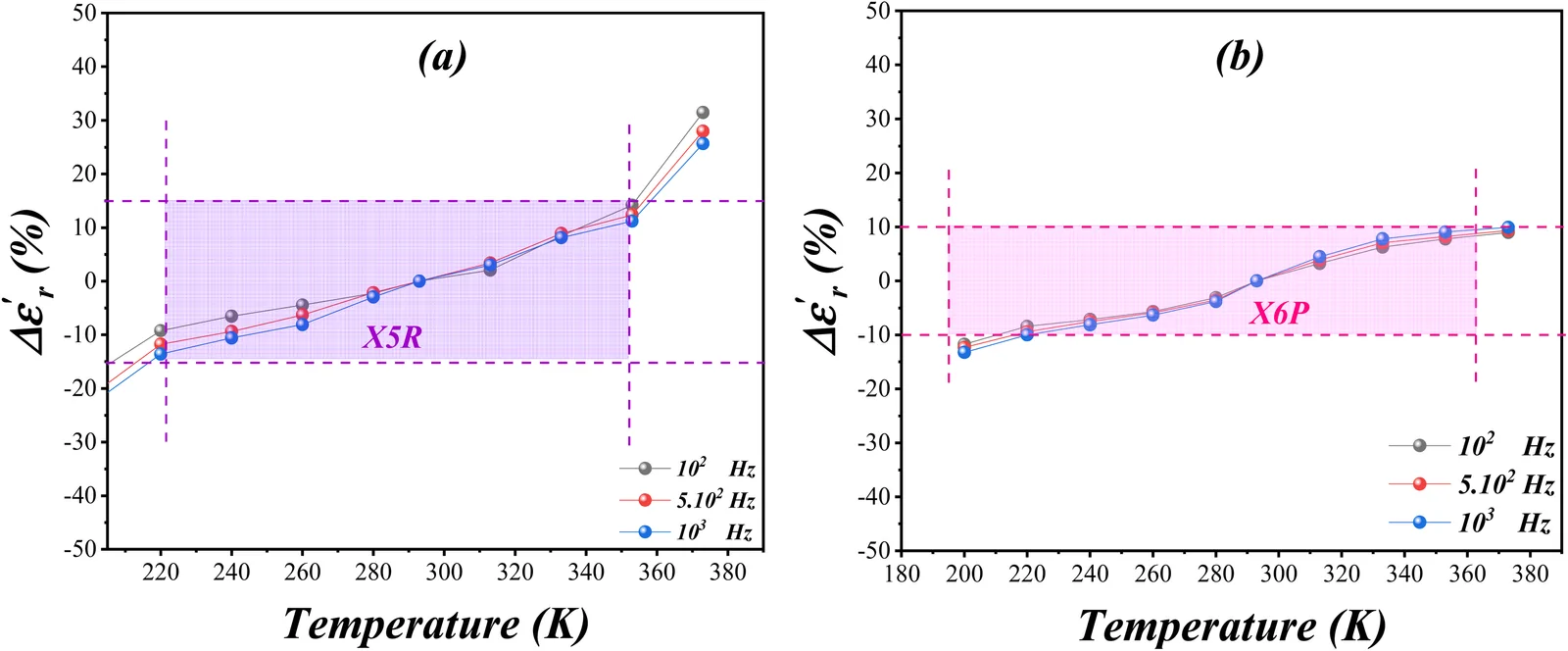
Temperature dependence of dielectric permittivity variation (
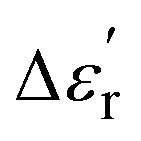
) at selected frequencies for (a) Sm_2_CuO_4_ under X5R limits (±15%), and (b) Sm_1.6_Nd_0.4_CuO_4_ under X6P limits (±10%).

The strategic Nd-substitution successfully suppresses extreme temperature-induced dielectric fluctuations, upgrading the ceramic directly from an X5R to highly stable X6Pcommercial classification. Achieving this rigorous X6P thermal stability while simultaneously preserving colossal permittivity (
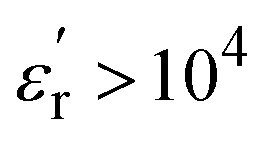
) and suppressing dielectric losses positions the prepared Sm_1.6_Nd_0.4_CuO_4_ ceramic as highly promising, next-generation candidate for advanced energy storage and high-performance microelectronic capacitor applications.

## 4. Conclusion

Single–phase tetragonal Sm_2−*x*_Nd_*x*_CuO_4_ (*x* = 0, 0.2, 0.4) ceramics were successfully synthesized *via* the conventional solid-state reaction method to systematically investigate the correlation between their structural modifications, charge transport kinetics, and dielectric performance. Rietveld refinement and microstructural analyses confirmed that substituting Sm^3+^ with the larger Nd^3+^ ion expands the crystal lattice, increases crystallite size, and promotes the formation of extanded monodomains witout introducing secondary phases. Furthermore, X-ray photoelectron spectroscopy revealed that this ionic size mismatch induces localized lattice strain, which subsequently increases the concentration of surface oxygen vacancies while preserving the expected oxidation states of the host and dopant ions.

The electrical transport kinetics of these perovskite ceramics exhibit a pronounced Negative Temperature Coefficient of Resistance (NTCR), with macroscopic conduction overwhelmingly governed by highly resistive grain boundaries. The direct current (DC) conductivity analysis uncovered a distinct thermal crossover near 290 K, where the dominant charge transport transitions from Variable range hopping at lower temperatures to Small polaron hopping at elevated temperatures, a process primarily mediated by oxygen vacancies. Notably, the optimized Sm_1.6_Nd_0.4_CuO_4_ composition demonstrates exceptional bolometric sensitivity, achieving a maximum Temperature Coefficient of Resistance of Approximately −5% at 390 K. this value significantly outperforms standard commercial uncooled bolometer materials, underscoring its potential for advanced thermal sensing technologies.

Beyond its transport properties, the highly substituted Sm_1.6_Nd_0.4_CuO_4_ ceramic exhibits a colossal permittivity (
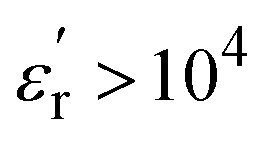
) coupled with moderate dielectric losses across intermediate frequencies. Complex modulus and impedance analyses confirmed that this immense charge storage capacity is driven by the IBLC effect and Maxwell–Wagner interfacial polarization. Crucially, the strategic incorporation of Neodymium effectively suppresses extreme temperature-induced dielectric fluctuations, upgrading the material's thermal stability from the pristine compound's X5R classification directly to the rigorous X6P commercial standard. By restricting the relative permittivity variation strictly within 
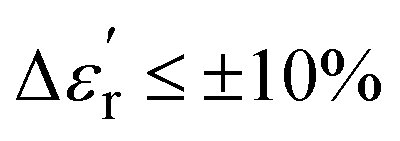
 over a broad operational window from −55 °C to 105 °C, the Sm_1.6_Nd_0.4_CuO_4_ ceramic emerges as a highly competitive and promising candidate for next-generation high-energy-density capacitors and advanced microelectronic applications.

## Conflicts of interest

The authors declare no conflicts of interest.

## Data Availability

The data that support the findings of this study are available from the corresponding author upon reasonable request.
